# The neuronal response at extended timescales: long-term correlations without long-term memory

**DOI:** 10.3389/fncom.2014.00035

**Published:** 2014-04-01

**Authors:** Daniel Soudry, Ron Meir

**Affiliations:** Department of Electrical Engineering, the Laboratory for Network Biology ResearchTechnion, Haifa, Israel

**Keywords:** neurons, temporal correlations, long memory, noise, input–output analysis, linear filters, power spectral density

## Abstract

Long term temporal correlations frequently appear at many levels of neural activity. We show that when such correlations appear in isolated neurons, they indicate the existence of slow underlying processes and lead to explicit conditions on the dynamics of these processes. Moreover, although these slow processes can potentially store information for long times, we demonstrate that this does not imply that the neuron possesses a long memory of its input, even if these processes are bidirectionally coupled with neuronal response. We derive these results for a broad class of biophysical neuron models, and then fit a specific model to recent experiments. The model reproduces the experimental results, exhibiting long term (days-long) correlations due to the interaction between slow variables and internal fluctuations. However, its memory of the input decays on a timescale of minutes. We suggest experiments to test these predictions directly.

## 1. Introduction

Long term temporal correlations, or “*f*^−α^ statistics” (Keshner, 1982), are ubiquitously found at multiple levels of brain and behavior (Ward and Greenwood, 2007, and refrences therein). For example, *f*^−α^ statistics appear in human cognition (Gilden et al., 1995; Repp, 2005), brain and network activity (measured using electroencephalograph or local field potentials Bédard et al., 2006, and refrences therin), and even Action Potentials (APs) generated by single neurons (Musha and Yamamoto, 1997; Gal et al., 2010). The presence of these long correlations in a neuron's AP responses suggests it is affected by processes with slow dynamics, which can retain information for long times. As a result, if these slow processes are also affected by APs, then the generation of each AP (indirectly) depends on a rather long history of the neuron's previous inputs and APs. This potentially allows a single neuron to perform complex computations over very long timescales. However, it remains unclear whether this type of computation indeed occurs.

Cortical neurons indeed contain processes taking place on multiple timescales. Many types of ion channels are known, with a large range of kinetic rates (Channelpedia, http://channelpedia.epfl.ch/). Additional new sub-cellular kinetic processes are being discovered at an explosive rate (Bean, 2007; Sjöström et al., 2008; Debanne et al., 2011). This variety is particularly large for very slow processes (Marom, 2010). Such rich biophysical machinery can potentially modulate the generation of APs on long timescales. Evidence for such abilities was observed in recent works, which investigated how cortical neurons temporally integrate noisy current stimuli (Lundstrom et al., 2008, 2010; Pozzorini et al., 2013). The temporal integration of the input was approximated using filters with power law decay, reflecting “long memory.” However, these filters were fitted only up to a timescale of about 10 s (or equivalently, frequencies smaller than 10^−1^ Hz), possibly due to the limited duration of the experiments, which involve intracellular recording.

**Figure 1:**
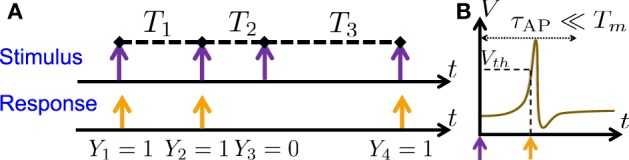
**Stimulation Regime. (A)** Stimulation consists of (extracellular) sparse current spikes, with inter-stimulus intervals *T*_*m*_ and Action Potential (AP) occurrences *Y*_*m*_. **(B)** An AP “occurred” if the voltage *V* crossed a threshold *V*_*th*_ following the (sparse) stimulus, with *T*_*m*_ ≫ τ_AP_.

This raises the question – would the neuron still have long memory on timescales longer than 10 s? Generally, the answer may depend on the type of stimulus used. For example, certain ion channels may “remember” non-sparse inputs longer than sparse inputs (Soudry and Meir, 2010). Here, we focus on the case of the sparse (AP-like) input (Figure 1), imitating the “natural” input for an axonal compartment which receives APs from a previous compartment. Such stimulation is used in various experiments (e.g., Grossman et al., 1979; De Col et al., 2008; Gal et al., 2010).

We find general conditions under which a neuron can generate *f*^−α^ statistics in its spiking activity, and show that this does not imply that a neuron has long memory of its history. Specifically, in order to generate *f*^−α^ statistics slow processes should span a wide range of timescales with slower processes having a higher level of internally generated fluctuations (e.g., more “noisy,” due to lower ion channel numbers). However, in a minimal model that generates this behavior, slow processes do not retain memory of the input fluctuations beyond a finite “short” timescale, even though they are affected by the membrane's voltage. A main reason for this is that the “fastest adaptation process” in the model adjusts the neuron's response in such a way that any perturbation in the response is canceled out, before slower processes are affected.

We fit the minimal model to the days-long experiments in Gal et al. (2010), where synaptically isolated individual neurons, from a rat cortical culture, were stimulated with extra-cellular sparse current pulses for an unprecedented duration of days. The neurons exhibited *f*^−α^ statistics, responding in a complex and irregular manner from seconds to days. The synaptic isolation of the neurons in the network, and their low cross-correlations indicate that these *f*^−α^ fluctuations are internally generated in the neurons (Appendix D). We are able to reproduce their results (Figure 3), and predict that the neuron should remember perturbations in its input for about 10^2^ s (Figure 4). We suggest further experiments to test these predictions (Figure 5).

The remainder of the paper is organized as follows. We begin in section 2.1 by presenting the basic setup. Then, in section 2.2, we present the general framework for biophysical modeling of neurons. Working in this framework, in section 2.3 we recall the mathematical formalism from Soudry and Meir (2014) and derive the power spectrum density for periodic input stimuli. Following a description of *f*^−α^ behavior in section 3.1, we provide in section 3.2 both general and “minimal” conditions for a neuron to display such scaling. In section 3.3 we consider the implications of the model for the input–output relation of the neuron, given general stationary inputs. In section 3.4 we demonstrate this numerically in a specific biophysical model which is fitted to the experimental results of Gal et al. (2010). We conclude in section 4 with a summary and discussion of our results. An extensive appendix contains many of the technical details used throughout the paper (see Supplementary Material).

## 2. Methods

### 2.1. Preliminaries

In our notation 〈·〉 is an ensemble average, *i* ≜ $\sqrt{-1}$, a non-capital boldfaced letter **x** ≜ (*x*_1_, …, *x*_*n*_)^⊤^ is a column vector [where (·)^⊤^ denotes transpose], and a boldfaced capital letter **X** is a matrix (with components *X*_*mn*_).

#### 2.1.1. Stimulation

As in Soudry and Meir (2014) we examine a single, synaptically isolated, excitable neuron under “spike” stimulation. In this stimulation regime, the stimulation current, *I*(*t*), consists of a train of identical short pulses arriving at times *t*_*m*_ and amplitude *I*_0_. The intervals between the stimulation times are denoted *T*_*m*_ ≜ *t*_*m* + 1_ − *t*_*m*_ (Figure 1A, *top*). We assume that the stimulation is sparse, i.e., *T*_*m*_ ≫ τ_AP_, with τ_AP_ being the timescale of an AP (Figure 1B). Since the neuron is “excitable” it does not generate APs unless stimulated, as in Gal et al. (2010) (i.e., the neuron is neither oscillatory nor spontaneously firing). However, after a stimulation the neuron can either respond with a detectable AP or not respond. We denote AP occurrences a *Y*_*m*_, where *Y*_*m*_ = 1 if an AP occurred immediately after the *m*-th stimulation, and 0 otherwise (Figure 1A, *bottom*). Note also that *Y*_*m*_ is not generally the same as the common count process generated from the APs by binning them into equally sized bins (Appendix B.1) – unless *T*_*m*_ is constant and equal to the bin size.

#### 2.1.2. Statistics

We assume both *Y*_*m*_ and *T*_*m*_ are wide-sense stationary (Papoulis and Pillai, 1965). We denote p_*_ ≜ 〈 *Y*_*m*_ 〉 to be the mean probability to generate an AP and T_*_ ≜ 〈 *T*_*m*_ 〉 as the mean stimulation period. Furthermore, we denote *Ŷ*_*m*_ ≜ *Y*_*m*_ − p_*_ and $\hat{T}$_*m*_ = *T*_*m*_ − T_*_ as the perturbations of *Y*_*m*_ and *T*_*m*_ from their means. An important tool in quantifying the statistics of signals is the power spectral density (PSD), namely the Fourier transform of the auto-covariance (Papoulis and Pillai, 1965). For analytical convenience, in this work we will use a PSD of the form

(1) $$S_{Y}\left(f\right)≜T_{*}\sum_{k\;=-\;\infty }^{\infty }\langle {\hat{Y}}_{m}{\hat{Y}}_{m\;+\;k}\rangle e^{-2\pi fT_{*}ik},$$

with 0 ≤ *f* ≪ *T*^−1^_*_ in Hertz frequency units. Note that this PSD is proportional to the PSD of the common binned AP (Equation 70), under periodical stimulus and for low frequencies – which is the regime under which we will investigate the PSD (similarly to the experiment Gal et al., 2010). We similarly define the PSD

(2) $$S_{T}\left(f\right)≜T_{*}\sum_{k\;=-\;\infty }^{\infty }\langle {\hat{T}}_{m}{\hat{T}}_{m\;+\;k}\rangle e^{-2\pi fT_{*}ik},$$

and the cross-PSD

(3) $$S_{YT}\left(f\right)≜T_{*}\sum_{k\;=-\;\infty }^{\infty }\langle {\hat{Y}}_{m}{\hat{T}}_{m\;+\;k}\rangle e^{-2\pi fT_{*}ik}.$$

### 2.2. General framework

We model the neuron in the standard framework of biophysical neural models – i.e., Conductance Based Models (CBMs). However, rather than focusing only on a specific model, we establish general results about a broad class of models. In this framework, the voltage dynamics of an isopotential neuron are determined by ion channels, protein pores which change conformations stochastically with voltage-dependent rates (Hille, 2001). On the population level, such dynamics are generically very well described by models of the form of Soudry and Meir (2014), (Equations 4–6)

(4) $$\dot{V}=f\left(V,r,s,I\left(t\right)\right)$$

(5) $$\dot{r}=A_{r}\left(V\right)r-b_{r}\left(V\right)+B_{r}\left(V,r\right)\xi_{r}$$

(6) $$\dot{s}=A_{s}\left(V\right)s-b_{s}\left(V\right)+B_{s}\left(V,s\right)\xi_{s}$$

with **voltage** *V*, stimulation current *I*(*t*), **rapid** variables **r** (e.g., *m*, *n*, *h* in the Hodgkin–Huxley (HH) model Hodgkin and Huxley, 1952), **slow** “excitability” variables **s** ∈ [0, 1]^*M*^ (e.g., slow sodium inactivation Chandler and Meves, 1970), white noise processes ξ_*r*/*s*_ (with zero mean and unit variance). Also, the matrices **A**_*r*/*s*_ and the vectors **b**_*r*/*s*_ can be written explicitly using the kinetic rates of the ion channels, while the matrices **B**_*r*/*s*_ can be written using those rates in addition to ion channel numbers. Lastly, we denote

$$D_{r}≜B_{r}B_{r}^{⊤};D_{s}≜B_{s}B_{s}^{⊤}$$

as the diffusion matrices (Orio and Soudry, 2012). In these models the voltage and the rapid variables constitute the AP generation, while the slow variables modulate the excitability of the cell. For simplicity, we assumed that **r** and **s** are not coupled directly, but this is non-essential (Soudry and Meir, 2014). The parameter space can be constrained (Soudry and Meir, 2012), since we consider here only *excitable*, non-oscillatory neurons which do not fire spontaneously^1^ and which have a single resting state – as is common for *isolated* cortical cells, e.g., Gal et al. (2010).

### 2.3. The power spectral density of the response

PSD-based estimators are central tools in quantifying long term correlations (Robinson, 2003; Lowen and Teich, 2005), and are commonly used in experimental settings – as in Gal et al. (2010). Therefore, in this section we focus on the PSD of the neural response under sparse stimulation regime (section 2.1) of a CBM (section 2.2).

#### 2.3.1. Recap – previous mathematical results

Typically, CBMs (Equations 4–6) contain many unknown parameters, and are highly non-linear. Therefore, it is quite hard to fit them using a purely simulation based approach, especially over long timescales, where simulations are long and models have more unknown parameters. Therefore, we developed a reduction method that simplifies analysis and enables fitting of such models. We refer the reader to Soudry and Meir (2014) for full mathematical details.

In this method, we semi-analytically^2^ reduce the full model (Equations 4–6) to a simplified model, under the assumption that the timescales of rapid and slow variables are well separated. Given another assumption, that the neuron dynamics are sufficiently “noisy,” we can linearize the model dynamics, so that (Soudry and Meir, 2014, Equation 12)

(7) $${\hat{Y}}_{m}=w^{⊤}\left(s\left(t_{m}\right)-s_{*}\right)+e_{m},$$

where *e*_*m*_ is a white-noise signal with zero mean and variance σ^2^_*e*_ ≜ p_*_ − *p*^2^_*_ (recall p_*_ is the mean probability to generate an AP) and s_*_ (the excitability fixed point) and *w*_*j*_ (an “effective weight” of component *s*_*j*_) can be found self-consistently together with p_*_ as a function of T_*_ (Soudry and Meir, 2014, Equation 10). After these quantities are found, an expression for the output PSD *S*_*Y*_ (*f*) in this model can be written explicitly. We let *X*_+_, *X*_−_ and *X*_0_ denote the averages of the quantity *X*_*s*_ during an AP response, a failed AP response and rest, respectively. Also, we denote

$$X_{*}≜\tau_{\text{AP}}T_{*}^{\;-1}\left(p_{*}X_{+}+\left(1-p_{*}\right)X_{-}\right)+\left(1-\tau_{\text{AP}}T_{*}^{\;-1}\right)X_{0}$$

as the steady state mean value of *X*_*s*_ [this would be *X*(p_*_, T_*_) in Equation 7 in Soudry and Meir, 2014]. For example, A_*_ and D_*_ are the respective steady state means of **A**_*s*_ and **D**_*s*_. Additionally, we denote (definition below Equation 12 in Soudry and Meir, 2014)

(8) $$a≜\tau_{\text{AP}}\left(\left(A_{+}-A_{-}\right)s_{*}-\left(b_{+}-b_{-}\right)\right)$$

as a “feedback” vector (see Figure 1C in Soudry and Meir (2014) to understand this interpretation), and (Soudry and Meir, 2014, Equation 14)

(9) $$H_{c}\left(f\right)≜{\left(2\pi fiI-A_{*}-T_{*}^{-1}aw^{⊤}\right)}^{-1}$$

as the “closed loop transfer function” (including the effect of the feedback), with **I** being the identity matrix. Using the above notation, we can derive the PSD of the response. Given a periodical stimulation ($\hat{T}$_*m*_ = 0) we obtain (Soudry and Meir, 2014, Equation 13)

(10) $$\begin{array}{l} S_{Y}(f)=w^{⊤}H_{c}(-f)D_{*}H_{c}^{⊤}(f)w\\ \;+T_{*}\sigma_{e}^{2}{\left|1+\;T_{*}^{-1}w^{⊤}H_{c}(-f)a\right|}^{2}. \end{array}$$

Though Equation (10) relies on two simplifying assumptions, extensive numerical simulations (Soudry and Meir, 2014, Figures 3–5) showed that this expression is rather robust and remains accurate in many cases even if these assumptions do not hold. Therefore, in this work we will always assume that Equation (10) is accurate.

#### 2.3.2. The effect of feedback

In the neuron, the slow excitability variables **s** affect the response of the neuron, which, in turn, affects the dynamics of the slow excitability variables. To simplify analysis, it is desirable to “isolate” this feedback effect. In order to do this, we apply the Sherman Morrison lemma to Equation (9),

$$w^{⊤}H_{c}\left(-f\right)=w^{⊤}H_{o}\left(-f\right){\left(1-T_{*}^{-1}w^{⊤}H_{o}\left(-f\right)a\right)}^{-1},$$

with

(11) $$H_{o}\left(f\right)≜{\left(2\pi fiI-A_{*}\right)}^{-1}$$

being the “open loop” version of **H**_*c*_ (*f*) (i.e., if ***a*** is set to zero). Using this in Equation (10) we obtain

(12) $$S_{Y}\left(f\right)=S_{Y}^{o}\left(f\right){\left|\kappa \left(f\right)\right|}^{-2},$$

with *S*^*o*^_*Y*_ (*f*) being the “open loop” version of *S*_*Y*_ (*f*) (i.e., *S*_*Y*_ (*f*) with ***a*** set to zero),

(13) $$S_{Y}^{o}\left(f\right)≜T_{*}\sigma_{e}^{2}+w^{⊤}H_{o}\left(-f\right)D_{*}H_{o}^{⊤}\left(f\right)w$$

and κ (*f*) determines the effect of the feedback

(14) $$\kappa \left(f\right)≜1-T_{*}^{-1}w^{⊤}H_{o}\left(-f\right)a.$$

Note that *κ* (*f*) depends on the feedback through the variable **a**. If **a** → 0, for example, the kinetic rates of **s** are not sensitive to AP occurrences^3^. In that case *κ* (*f*) → 1 and *S*_*Y*_ (*f*) → *S*^*o*^_*Y*_ (*f*).

#### 2.3.3. Partial fractions decomposition

In order to simplify analysis, we decompose the vector expressions in Equations (13, 14) to partial fractions.

If A_*_ is diagonalizable, than we can write Equation (13) as (Appendix A.1)

(15) $$S_{Y}^{o}\left(f\right)=T_{*}\sigma_{e}^{2}+\sum_{k\;=\;1}^{M}\frac{c_{k}}{{\left(2\pi f\right)}^{2}+\lambda_{k}^{2}},$$

where the poles λ_*k*_ are the inverse timescales of the slow variables (the eigenvalues of A_*_), arranged from large to small according to their magnitudes (0 < |λ_*M*_| < |λ_*M* − 1_| < … < |λ_1_|) and

(16) $$c_{k}=\sum_{j\;=\;1}^{M}w_{k}D_{kj}w_{j}\frac{2\lambda_{k}}{\lambda_{k}+\lambda_{j}}$$

being the amplitude of these poles, with *D*_*kj*_ and *w*_*k*_ being the respective components of D_*_ and **w** in a basis in which A_*_ is diagonal. Note that, ∀*k*, Re [λ_*k*_] < 0 (from the properties of A_*_).

Using a similar derivation for κ (*f*), we obtain

(17) $$\kappa \left(f\right)=1-\sum_{k\;=\;1}^{M}\frac{T_{*}^{-1}w_{k}a_{k}}{2\pi fi-\lambda_{k}},$$

with *a*_*k*_ and *w*_*k*_ being the respective components of **a** and **w** in a base in which A_*_ is diagonal.

#### 2.3.4. Example – a “diagonal” model

For concreteness, we demonstrate our results on a simple model in which A_*_ is a diagonal matrix and, as a result, D_*_ (which depends on A_*_) is also diagonal. In this “diagonal” model all the components of **s** are uncoupled (i.e., belong to different channel types), Equation (6) can be written as (Soudry and Meir, 2014, section 4.1)

(18) $${\dot{s}}_{k}=\delta_{k}\left(V\right)\left(1-s_{k}\right)-\gamma_{k}\left(V\right)s_{k}+\sigma_{s,k}\left(V,s_{k}\right)\xi_{s,k}$$

∀ *k*∈{1, …, *M*}, where σ_*s*,*k*_(*V*, *s*) = [(δ_*k*_(*V*)(1 − *s*_*k*_) + γ_*k*_(*V*)*s*_*k*_) *N*^ − 1^_*s*,*k*_]^1/2^ and *N*_*s*,*k*_ are the number of slow ion channels of type *k*. Similarly as before, γ_+,*k*_,γ_−,*k*_ and γ_0,*k*_ denote the averages of the kinetic rate γ_*k*_(*V*) during an AP response, a failed AP response and rest, respectively. In addition

$$\gamma_{*,k}\;=\;\tau_{\text{AP}}T_{*}^{-1}\left(p_{*}\gamma_{+,k}+\left(1-p_{*}\right)\gamma_{-,k}\right)+\left(1-\tau_{\text{AP}}T_{*}^{-1}\right)\gamma_{0,k}$$

is the average γ_*k*_(*V*) in steady state. We use a similar notation for δ. Therefore

$$\begin{array}{l} {\left(A_{*}\right)}_{kk}=-\gamma_{*,k}-\delta_{*,k}\\ {\left(D_{*}\right)}_{kk}\;=\;\frac{1}{N_{s,k}}\frac{\gamma_{*,k}\delta_{*,k}}{\gamma_{*,k}+\delta_{*,k}} \end{array}$$

with zero on all other (non-diagonal) components and

(19) $$a_{k}=\tau_{AP}\frac{\left(\gamma_{*,k}\left(\delta_{+,k}-\delta_{-,k}\right)-\left(\gamma_{+,k}-\gamma_{-,k}\right)\delta_{*,k}\right)}{\gamma_{*,k}+\delta_{*,k}}.$$

Therefore, in Equations (15, 16) we have,

(20) $$\lambda_{k}=-\gamma_{*,k}-\delta_{*,k}$$

(21) $$c_{k}=w_{k}^{2}D_{kk}=\frac{w_{k}^{2}}{N_{s,k}}\frac{\gamma_{*,k}\delta_{*,k}}{\gamma_{*,k}+\delta_{*,k}}.$$

Importantly, by tuning the parameters *M*, γ_*k*_(*V*), δ_*k*_(*V*), *N*_*s*,*k*_ and *w*_*k*_ we seem to have complete freedom in determining λ_*k*_, *c*_*k*_ and *a*_*k*_ (Equations 19–21). This, in turn, would give complete freedom in tuning *S*^*o*^_*Y*_ (*f*) and κ (*f*). Therefore, it seems that for any CBM (i.e., not only diagonal models) we can find an *equivalent* diagonal model – which produces exactly the same PSD of the response.

The only caveat in the previous argument is that in non-diagonal models λ_*k*_ can be complex, but not in a diagonal model, since the kinetic rates γ_*k*_(*V*) and δ_*k*_(*V*) must be real numbers. How would the situation change if some of the poles had complex values? Complex poles (i.e., for which Im[λ_*k*_] > 0) always come in conjugate pairs. These pairs behave asymptotically (i.e., for 2π *f* ≫ |λ_*k*_| or 2π *f* ≪ |λ_*k*_|) very similarly to two real poles, with an additional “resonance” (either a bump or depression) in a narrow range in the vicinity of these poles (i.e., 2π *f* ~ |λ_*k*_|) (see Appendix A.2, or Oppenheim et al., 1983).

## 3. Results

### 3.1. Background on *f*^−α^ statistics

As observed in Gal et al. (2010), the responses of isolated neurons exhibit long-term correlations robustly ^4^, under sparse pulse stimulation (Figure 1 and section 2.1). Signals with such long-term correlations are often described by the term “*f*^−α^ noise.” This is because the Power Spectral Density (PSD, Papoulis and Pillai, 1965) is a central tool in detecting and quantifying such signals (Robinson, 2003; Lowen and Teich, 2005). As the name implies, if the AP pattern *Y*_*m*_ is a “*f*^−α^ noise signal” then its PSD (Equation 10) has a *f*^−α^ shape

(22) $$S_{Y}\left(f\right)\propto f^{-\alpha },$$

where the PSD is defined here as in Equation (1). As is usually the case for most *f*^ − α^ phenomena, Equation (22) is true only in a certain range *f*_min_ ≤ *f* ≤ *f*_max_, and with 0 < α ≤2. Note also that if α > 1, then *f*_min_ > 0 necessarily^5^. Such *f*^−α^ behavior is considered interesting due to its “scale-free” properties, which can sometimes indicate a “long memory,” as explained in the introduction. Therefore, it is interesting to ask the following questions:

1. What is the biophysical origin of the *f*^−α^ behavior?
2. Does this *f*^−α^ behavior imply that the neuron “remembers” its history on very long timescales (hours and days)?

We aim to answer the first question in section 3.2, focusing on the case of periodical stimulation *T*_*m*_ = T_*_, as in Gal et al. (2010). The second question is addressed in section 3.3, where we examine a general sparse stimulation process *T*_*m*_. Finally, in section 3.4.2 we fit a specific CBM (which is an extension of a previous CBM) so it adheres to this set of minimal constraints. We numerically reproduce the experimental results of Gal et al. (2010) and demonstrate our predictions.

### 3.2. Biophysical modeling of *f*^−α^ statistics

As we explained in the introduction, neurons contain a large variety of processes operating on slow timescales. These processes are, in many cases, not well characterized or contain unknown parameters. Therefore, it is hard to model the behavior of the neuron on slow timescales with a CBM using only simulation. With so many unknowns, an exhaustive parameter search is unfeasible ^6^. Fortunately, since we derived a semi-analytic expression for the PSD (Equation 10), starting from some initial “guess” (as to which process to include, and with what parameters), it is relatively straightforward to tune the parameters so that the CBM reproduces the experimental results (i.e., by maximizing some “goodness of fit” measure).

However, even if a specific model could be found to reproduce the experimental results, it would still be unclear whether or not this is would be a “useful” model – one which can be used to infer the biophysical properties of the neuron, or its response to untested inputs. The first problem is that CBMs are highly degenerate, where different parameter values can generate similar behaviors^7^, so we can never be sure if the “correct” model was inferred. The second problem is that it is unclear whether a “correct” model would be generally useful – since different neurons from the same type can have very different parameters (Marder and Goaillard, 2006).

In order to address the first problem, initially, in section 3.2.1 we analyze Equation (10), and attempt to answer a more general question – what class of CBM models can generate the experimental results? We find “rather general” sufficient conditions – i.e., which, given a few assumptions, also become necessary conditions. Next, in section 3.2.2, we aim to find a “minimal” set of constraint on a CBM to fulfill theses conditions. Qualitatively, these conditions indicate that, in order to reproduce the experimental results, a general CBM must:

1. Include only a finite number of ion channels of each type (implying a stochastic model).
2. Include few slow processes with timescales “covering” the range of timescales over which *S*_*Y*_ (*f*) ∝ *f*^−α^ is observed.
3. Obey a certain scaling relation (with an exponent of 1−α), implying that slower processes are more “noisy.”

More detailed explanations of these conditions, and a concrete example, are provided in the following two subsections.

#### 3.2.1. General conditions for *f*^−α^ statistics

In this section we derive general conditions on the parameters of a CBM (section 2.2) so it can generate robust *f*^−α^ statistics in *S*_*Y*_ (*f*). Here, we focus on the case of sparse periodical input *T*_*m*_ = T_*_ ≫ τ_AP_ (as in Gal et al., 2010).

This analysis is based on the decomposition of the PSD *S*_*Y*_ (*f*) as a ratio of *S*^*o*^_*Y*_ (*f*) and the feedback term |κ (*f*)|^2^. Recall that *S*_*Y*_ (*f*) ∝ *f*^−α^ is robustly observed for all stimulation parameters – even when p_*_ is near 0 or 1 (see section 3.1). Note that one can arbitrarily vary p_*_ by changing the stimulation parameters (such as *I*_0_ or T_*_). It is straightforward to show that when p_*_ → 0 or p_*_ → 1, the effect of feedback is negligible^8^, and therefore *S*_*Y*_ (*f*) ≈ *S*^*o*^_*Y*_ (*f*). This implies that, at least for some simulation parameters, *S*^*o*^_*Y*_ (*f*) ∝ *f*^−α^. For this reason, and for the sake of analytical simplicity, we first develop general conditions so that *S*^*o*^_*Y*_ (*f*) ∝ *f*^−α^, and later we discuss the effects of the feedback κ (*f*).

Note from Equation (15) that if *M* (the dimension of **s** – the number of slow processes) is finite, one can have *S*^*o*^_*Y*_ (*f*) ∝ *f*^−α^ exactly if and only if α = 0 or 2. However, these values are far from what was measured experimentally (Equation 42). Therefore, *S*^*o*^_*Y*_ (*f*) ∝ *f*^−α^ can be generated exactly only in some limit (in which *M* is infinite), or approximately (if *M* is finite). Also, note that if 2π *f* ≫|λ_1_|, then *S*^*o*^_*Y*_ (*f*) − T_*_ σ^2^_*e*_ ∝ *f*^−2^. Additionally, if 2π *f* ≪ |λ_*M*_|, we have *S*^*o*^_*Y*_ (*f*) ≈ constant. Therefore, Equation (15) can generate *S*^*o*^_*Y*_ (*f*) ∝ *f*^−α^ with 0 < α < 2 only for |λ_*M*_| < 2π *f* < |λ_1_|.

Next, we explain when this becomes possible. For simplicity assume that in Equation (15) T_*_ σ^2^_*e*_ is negligible and all the poles are real (the effect of complex poles will be discussed below). We define the following pole density

(23) $$\rho \left(\lambda \right)≜\sum_{k\;=\;1}^{M}c_{k}\delta \left(\lambda -\lambda_{k}\right)$$

where δ(·) is Dirac's delta function. Using Equations (15 and 23) we obtain

(24) $$S_{Y}^{o}\left(f\right)=\int \frac{\rho \left(\lambda \right)d\lambda }{{\left(2\pi f\right)}^{2}+\lambda^{2}}.$$

For |λ_*M*_| ≪ 2π *f* ≪ |λ_1_| and 0 < α < 2, Equation (24) becomes

(25) $$S_{Y}^{o}\left(f\right)=Cf^{-\alpha }$$

if and **only** if (Appendix A.3)

(26) $$\rho \left(\lambda \right)=\rho_{0}{\left|\lambda \right|}^{1\;-\;\alpha }$$

in the range |λ_1_| > |λ| > |λ_*M*_|, with *ρ*_0_ = 2π^−1^*C* sin(πα/2). Therefore, *ρ*(λ), the distribution of the poles, must scale similarly to *S*^*o*^_*Y*_ (*f*) (but with a different exponent).

Several comments are in order at this point.

**Figure 2:**
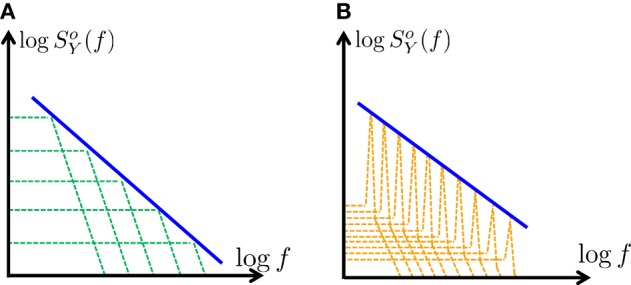
**Generating *f*^-α^ PSD using a finite number of poles – a graphic description**. Using partial fraction decomposition (Equation 15) *S*^*o*^_*Y*_ (*f*) ∝ *f*^-α^ (blue) can be approximated (on a log–log scale) in two distinct ways: **(A)** Using a sum of a real poles (green), with scaled amplitudes (approximating Equation 26) **(B)** Using a sum of complex poles (orange), with scaled “resonance peaks” (Equation 46). In this work we focus on the first case **(A)**, since it is simpler and requires far fewer poles.

1. It was previously known that, in a linear system, a *f*^−α^ PSD could be generated using a similarly scaled sum of real poles (Keshner, 1979, 1982). The novelty here is two-fold: (1) Quantitatively analyzing the PSD of CBMs (which are highly non-linear) in a similar way (through Equation 10) (2) Finding that condition 26 is not only sufficient, but necessary.
2. Formally, Equation (26) can be exact only in the continuum limit where the number poles is infinite and they are closely packed. However, in practice, Equation (25) remains a rather accurate approximation even if the poles are few and well separated (Figure 2A), as we shall demonstrate in the next section (as in Keshner, 1979, 1982). Clearly, for simulation purposes, it is beneficial to use a CBM with a finite number of (preferably, few) poles.
3. We have assumed that all the poles are real. What happens if some of the poles are complex? Recall (section 2.3.4) that if some poles have complex values then *S*^*o*^_*Y*_ (*f*) also has “resonances” (bumps or depressions) in a narrow range near these poles. Technically, scaling these resonance peaks can also be used to approximate Equation (25) (Figure 2B). However, we did not pursue this method here since it would require significantly more poles and would be much harder to implement.
4. Note that so far we have discussed only *S*^*o*^_*Y*_ (*f*). One can perform a similar analysis directly on *S*_*Y*_ (*f*). However, we find it is easier to first simplify κ (*f*) and then use Equation (12). From Equation (12) the PSD *S*_*Y*_ (*f*) will have a power-law shape in the range |λ_*M*_|≪2π *f* ≪ |λ_1_| if, in that range: either (1) the magnitude of κ (*f*) is constant or slowly varying, or (2) κ (*f*) also has a power-law shape. In the first case the exponent of *S*_*Y*_ (*f*) will be the same as the exponent of *S*^*o*^_*Y*_ (*f*), and in the second case the exponent will differ. The conditions for both cases can be derived similarly to our analysis of *S*^*o*^_*Y*_ (*f*). We demonstrate this next, in a more specific context.

#### 3.2.2. A minimal model for *f*^−α^ statistics

In the previous section we found general conditions under which Equation (13) gives *S*^*o*^_*Y*_ (*f*) ∝ *f*^−α^. In this section, we aim is to generate *S*^*o*^_*Y*_ (*f*) ∝ *f*^−α^ over *f*_min_ < *f* < *f*_max_ in a minimal model, in which *M* (the dimension of **s**) is as small as possible. As explained in section 2.3.4 we do not lose any relevant generality if we restrict ourselves to the case where A_*_ is diagonal (Equation 18). From Equation (26), we know that |λ_*k*_| must “cover” the frequency range *f*_min_ < *f* < *f*_max_. In order for *M* to be small, we choose λ_*k*_ to be uniform over a logarithmic scale (similarly to Keshner, 1982), so λ_*k*_ ∝ ϵ^*k*^ with ϵ < 1. The “simplest” way to achieve this is to have (see Equation 18)

(27) $$\gamma_{k}\left(V\right)=\gamma_{1}\left(V\right)\epsilon^{k\;-\;1}\;;\;\delta_{k}\left(V\right)=\delta_{1}\left(V\right)\epsilon^{k\;-\;1}$$

so

(28) $$\lambda_{k}=\lambda_{1}\epsilon^{k\;-\;1}.$$

In order for λ_*k*_/(2π) to cover the range [*f*_min_,*f*_max_] we require that

(29) $$\left|\lambda_{1}\right|>2\pi f_{\text{max}};\left|\lambda_{M}\right|=\left|\lambda_{1}\right|\epsilon^{M\;-\;1}\ll 2\pi f_{\text{min}}.$$

Given *M*, this sets a constraint on ϵ. In order to have scaling in ρ(λ), as in Equation (26), we also require that *c*_*k*_ ∝ |λ |^1−α^ *d*λ ∝ ϵ^(2−α)*k*^, since *d* λ = λ_*k*_ − λ_*k* − 1_ ∝ ϵ^*k*^. Therefore, from Equations (21) and (20) we have

$$\frac{w_{k}^{2}}{N_{s,k}}\propto \epsilon^{\left(1-\alpha \right)k}.$$

so that *S*^*o*^_*Y*_ (*f*) ∝ *f*^−α^. Therefore, we require that *w*_*k*_ ∝ ϵ^−μ *k*^, *N*_*s*,*k*_ ∝ ϵ^*νk*^ with 2μ + *ν* = α − 1. For μ > 0 the slower processes (larger *k*) have larger weight. For *ν* > 0 slower processes have a smaller number of ion channels (therefore, they are more “noisy”).

In Appendix A.4, we investigate what type of scaling will generate also *S*_*Y*_ (*f*) ∝ *f*^−α^, taking into account the effects of feedback (through κ (*f*)). We conclude that, because of the feedback, a value of μ > 0 would not change the exponent of *S*_*Y*_ (*f*) over a “reasonable” range of parameters (i.e., assuming *ν* > −2). Therefore, the simplest way to generate *S*_*Y*_ (*f*) ∝ *f*^−α^ would be to take μ = 0. In this case, we have (Equation 59), for −1 < *ν* < 1 and |λ_*M*_| ≪ 2π *f* ≪ |λ_1_|,

(30) $$S_{Y}\left(f\right)\propto \frac{1}{N_{s,1}}\frac{f^{-\left(1+\nu \right)}}{\ln^{2}f},$$

where the logarithmic correction arises from the effect of feedback κ (*f*). A few comments on Equation (30) are in order at this point.

1. Due to the logarithmic correction, in order to approximate *S*_*Y*_ (*f*) ∝ *f*^−α^ it is a reasonable choice to set *ν* slightly higher than α−1, e.g.,
  (31) ν=α−0.9.
2. Even if there is no scaling in the parameters (i.e., μ = *ν* = 0), we obtain *S*_*Y*_ (*f*) ∝ *f*^−1^ (neglecting logarithmic factors).
3. Equation (30) is based on asymptotic derivation, which is correct in two opposing limits (“sparse” or “dense” poles, Appendix A.5), indicating that these results are rather robust to parameter perturbations.
4. The magnitude of the ion channels number *N*_*s*,1_ is inversely proportional to the magnitude of *S*_*Y*_ (*f*) (i.e., its proportionality constant), while the value *w*_1_ (the magnitude of the weights) does not affect *S*_*Y*_ (*f*).
5. When *N*_*s*,1_ → ∞ we have *S*_*Y*_ (*f*) → 0, implying that in the deterministic limit, such a CBM does not generate *f*^−α^ noise (in accordance with our results from Soudry and Meir, 2012).

### 3.3. The input–output relation of the neuron

In the previous section we derived minimal biophysical constraints under which a neuron may generate *f*^−α^ statistics in response to periodic stimulation. In this section we explore the input–output relation of the neuron under these constraints, in the case where the inter-stimulus intervals *T*_*m*_ form a general (sparse) random process. We decompose the neuronal response into contributions from its “long” history of internal fluctuations and its “short” history of inputs, quantifying neuronal memory.

#### 3.3.1. The linearized input–output relation

Recall that $\hat{T}$_*m*_ ≜ *T*_*m*_ − T_*_, with T_*_ ≜ 〈 *T*_*m*_ 〉 and *S*_*T*_ (*f*) the PSD of *T*_*m*_ (Equation 2). As explained in Soudry and Meir (2014), for a *general* CBM^9^ we can decompose *Ŷ*_*m*_, the fluctuations in the neuronal response, to a linear sum of the history of the input and internal noise, i.e.,

(32) $${\hat{Y}}_{m}=\sum_{k\;=\;0}^{\infty }h_{k}^{\text{ext}}{\hat{T}}_{m\;-\;k}+\sum_{k\;=\;0}^{\infty }h_{k}^{\text{int}}z_{m\;-\;k},$$

with the filter *h*^ext^_*k*_ used to integrate *external* fluctuations in the inputs, and the filter *h*^int^_*k*_ used to integrate *z*_*m*_, a zero mean and unit variance white noise representing *internal* fluctuations (e.g., ion channel noise). It is easier to analyze this I/O in the frequency domain, where Equation (32) becomes (Soudry and Meir, 2014, Equation 20)

(33) $$\hat{Y}\left(f\right)=H^{\text{ext}}\left(f\right)\hat{T}\left(f\right)+H^{\text{int}}\left(f\right)z\left(f\right),$$

where we define *X*(*f*) to be the Fourier transform of *X*(*t*). Together, *H*^ext^(*f*) and *H*^int^(*f*) describe the $\hat{T}$_*m*_ → *Ŷ*_*m*_ neuronal I/O at very long timescales.

Note that these filters are related to the PSDs, in the following way

(34) $$S_{YT}\left(f\right)=H^{\text{ext}}\left(-f\right)S_{T}\left(f\right),$$

(35) $$S_{Y}\left(f\right)={\left|H^{\text{ext}}\left(f\right)\right|}^{2}S_{T}\left(f\right)+{\left|H^{\text{int}}\left(f\right)\right|}^{2}$$

where we recall that *S*_*YT*_ (*f*) is the cross-PSD (Equation 3). Notably, from Equation (35), if the input to the neuron is not periodical (so, *S*_*T*_ (*f*) ≠ 0), then the PSD *S*_*Y*_ (*f*) should be the same as calculated previously, except for the addition of |*H*^ext^(*f*)|^2^*S*_*T*_ (*f*).

#### 3.3.2. The shape of the input–output filters

For a general CBM, we can derive semi-analytically the exact form of the filters in Equation (33) from its parameters, as we did for *S*_*Y*_ (*f*). For example, if $\hat{T}$_*m*_ = 0 (periodical input), then also *S*_*T*_ (*f*) = 0, and so

(36) $${\left|H_{\text{int}}\left(f\right)\right|}^{2}=S_{Y}\left(f\right),$$

where *S*_*Y*_ (*f*) is the PSD we derived previously (Equation 10). Additionally, we obtain (Soudry and Meir, 2014, Equation 17)

(37) $$H^{\text{ext}}\left(f\right)=T_{*}^{-1}w^{⊤}H_{c}\left(f\right)d.$$

with

(38) $$d≜A_{0}s_{*}-b_{0}.$$

Next, we find both filters for the minimal model described in section 3.2.2. Recall that in this model

(39) $$w_{k}=w_{1};a_{k}\propto \epsilon^{k};d_{k}\propto \epsilon^{k}$$

with *a*_1_ and *d*_1_, respectively given by Equations (8) and (38). To simplify analysis, we derive an asymptotic form for both filters, for the cases |λ_*M*_| ≪ 2π *f* ≪ |λ_1_| and 2π *f* ≫ |λ_1_|. First, from Equation (36), and Equation (59), we find

(40) $$\left|H_{\text{int}}\left(f\right)\right|\sim \{\begin{array}{l} f^{-\alpha /2}/\ln f,\text{if}\left|\lambda_{M}\right|\ll 2\pi f\ll \left|\lambda_{1}\right|\\ \text{constant},\text{if}2\pi f\gg \left|\lambda_{1}\right| \end{array}.$$

Similarly, from Equation (37), we find (Appendix A.6) that for the minimal model the interpolation between the two asymptotic cases is monotonic, so we can approximate

(41) $$H^{\text{ext}}\left(f\right)\approx \frac{qd_{1}}{2\pi fi-qa_{1}}.$$

where *q* ≜ (1 − ϵ)^−1^*T*^−1^_*_w_1_. A few comments on Equations (40, 41) are in order at this point.

1. We found that *H*^ext^(*f*) is a low pass filter with a pole at *f*_ext_ = *qa*_1_/2π while *H*^int^(*f*) ~ *f*^−α/2^ for 2π *f* ≪ |λ_1_|. Consequently, in the temporal domain (Equation 32), for large *t* (i.e., large *k*), the neuron's memory of its external input decays exponentially (*h*^ext^_*k*_ ~ *e*^−*f*^_1T_*_k_), while its memory of its internal fluctuations decays as a power law (*h*^int^_*k*_ ~ *k*^−(1−α/2)^). Therefore, the input memory has a finite timescale (equal to *f*^−1^_ext_), while the memory of internal fluctuations is “long” (with a cutoff only near *f*^−1^_min_).
2. It is perhaps surprising that Equation (37), which has multiple poles, becomes a low pass filter with a single pole *f*_1_. The derivation (Appendix A.6) gives two main reasons for this. First, the scaling of *w*_*k*_ and *d*_*k*_ in Equation (39) induces only a weak (logarithmic) scaling of the poles in open-loop. Second, even this weak scaling is canceled by the effects of the feedback.
3. Naturally, other models may have a different shape of *H*^ext^(*f*). This could be probed directly, as we explain later, in section 3.4.3.

### 3.4. Modeling experimental results

In this section we apply our results to experimental data, described in section 3.4.1. In section 3.4.2 we implement the set of “minimal constraints” we found in section 3.2.2 in a specific CBM, and fit it to experimental data in which *S*_*Y*_ (*f*) ∝ *f*^−α^. The analytical results in section 3.2 suggest that this specific CBM is a “reasonable” representative of the family of CBMs that can generate the experimental results. Other members of this family can be reached by varying the parameters within the (either minimal or general) constraints. Next, in section 3.4.3 we use our results from section 3.3.2 on the fitted model. We show that, although internal fluctuations in the model can affect the neural response on a timescale of days, the memory of the input is only retained for a duration of minutes. We suggest specific experiments to test this prediction. In section 3.4.4 we suggest further predictions

#### 3.4.1. Experimental details

The experiment from Gal et al. (2010), where a single *synaptically isolated* neuron, residing in a culture of rat cortical neurons, is stimulated periodically with a train of extracellular short current pulses with constant amplitude *I*_0_. The observed neuronal response was characterized by different modes (Gal et al., 2010, Figure 2). We focus on the “intermittent mode” steady state, in which 0 < p_*_ < 1 (i.e., sometimes the stimulation evokes an AP, and sometimes it does not). The patterns observed in *Y*_*m*_, the AP occurrences timeseries, are rather irregular (Gal et al., 2010, Figure 2E), span multiple timescales (Gal et al., 2010, Figure 5) and variable (i.e., patterns are not repeatable Gal et al., 2010, Figure 9A). More quantitatively, as indicated by the analysis (Gal et al., 2010, Figure 6), for *all* intermittently firing neurons, the patterns in *Y*_*m*_ fall into the category of “*f*^−α^ noise” where the value of α varied significantly between neurons – with

(42) α=1.43±0.35

(mean ± *SD*). As we explained in section 3.1, this *f*^−α^ behavior is true only in some limited range *f*_min_ < *f* < *f*_max_. From the experimental data, (Figure 6C in Gal et al., 2010) it can be estimated that *f*_min_ < 10^−5^Hz and *f*_max_ ~ 10^−2^Hz. Also, since α > 1, then 0 < *f*_min_ (see section 3.1).

#### 3.4.2. The HHMS model – a biophysical implementation of the minimal constraints

In our previous work (Soudry and Meir, 2012) we already fitted a model that fits many of the “mean” properties of the neuronal response (e.g., firing modes, transients and firing rate). This model is an extension of the original Hodgkin–Huxley model which includes Slow sodium inactivation (Chandler and Meves, 1970; Fleidervish et al., 1996) (The HHS model, see Appendix C.1). In order to maintain this fit with the experimental results, we extend the HHS model with additional slow components, obeying Equation (18). We denote this as the HHMS model (Hodgkin Huxley model with Many Slow variables, Appendix C.2). The equations are identical to the HHS model, except that in the voltage equation (Equation 73) *g*_*Na*_*s* is replaced by *g*_*Na*_*M*^−1^ $\sum_{i\;=\;1}^{M}s_{i}$, where *s*_1_ has the same equation as *s* in the HHS model (Equation 77). By symmetry, this gives identical weights to component *s*_*i*_ (i.e., ∀*k*: *w*_*k*_ = *w*_1_). The remaining rates (for *k* ≥ 2) are chosen according to our constraints, so γ_*k*_(*V*) = γ(*V*) ϵ^*k* − 1^, δ_*k*_(*V*) = δ(*V*) ϵ^*k* − 1^ (as in Equation 27), where γ(*V*) and δ(*V*) are taken from the HHS model (Equation 77) and also *N*_*s*,*k*_ = *N*_*s*_ ϵ^*ν k*^. Therefore, the only free parameters are ϵ, *M*, *N*_*s*_, *ν* and *I*_0_ (*I*_0_ is the current amplitude of the stimulation pulses).

**Figure 3:**
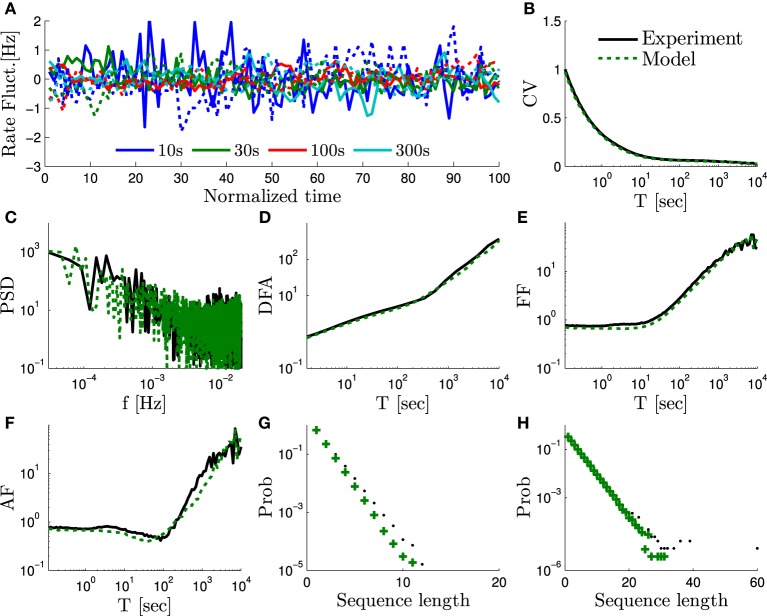
**The measures of “scale free” rate dynamics in the HHMS model – comparison of the experimental data from Gal et al. (2010) and a simulation of the extended HHS model (solid and dashed lines, respectively)**. We use here the same measures as in Figure 6 in Gal et al. (2010): **(A)** The firing rate fluctuations estimated using bins of different sizes (*T* = 10, 30, 100, and 300 s) and plotted on a normalized time axis (units in number of bins), after subtracting the mean of each series. **(B)** CV of the bin counts, as a function of bin size, plotted on a log-linear axis. **(C)** Firing rate periodogram. **(D)** Detrended fluctuations analysis. **(E)** Fano factor (FF) curve. **(*f*)** Allan factor (AF) curve. **(G)** Length distribution of spike–response sequences, on a half-logarithmic axes. **(H)** Length distribution of no-spike-response sequences, on a double-logarithmic axes. For additional details on measures used see Appendix B.1.

This model can be used to fit the experimental results for any α ∈ [0,2). We performed a numerical simulation of the full equations (Equations 4–6) of the HHMS model under periodical stimulation with T_*_ = 50 ms. We aimed to fit an experiment from Gal et al. (2010), which had a similar stimulation and exhibited *S*_*Y*_ (*f*) ~ *f*^−α^, with α = 1.4 (which is approximately the average α value measured in Gal et al., 2010). The current amplitude *I*_0_ was set to *I*_0_ = 7.7 μ *A* so that the model would have the same mean response probability p_*_ ≈ 0.4 as in the experimental data (using the self consistent equations for p_*_ from Soudry and Meir, 2014). We chose *M* = 5 and ϵ = 0.2 in order to satisfy constraint Equation (29) with a minimal *M*. We chose *ν* = 0.5 to satisfy Equation (31). Lastly, we chose *N*_*s*_ = 10^4^ in order to fit the magnitude of the *S*_*Y*_ (*f*). This reproduced all the scaling relations observed experimentally (Figure 3).

#### 3.4.3. Predictions – probing the input–output relation of the neuron

After fitting the HHMS model to the experimental results, we can examine its resulting linearized input–output relation, described by the filters *H*^ext^(*f*) and *H*^int^(*f*) (Equation 33). The *H*^int^(*f*) filter integrates internal fluctuations, while the *H*^ext^(*f*) filter determines how external fluctuations (in the input) affect its response.

**Figure 4:**
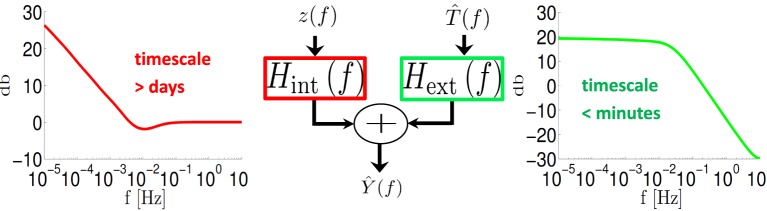
**System decomposition into external (input – $\hat{T}$(*f*)) and internal (fluctuation – *z*(*f*)) filters**. For a fitted HHMS model, *H*^ext^(*f*) is a low pass filter with cutoff <10^−2^ Hz while *H*_int_ (*f*) ~ *f*^-α/2^ for *f* < 10^−2^ Hz.

In accordance with the asymptotic forms in Equations (40) and (41), we find that *H*^ext^(*f*) is a low pass filter with a pole *f*_ext_ ~ 10^−2^ Hz (Figure 4, green) while *H*^int^(*f*) ~ *f*^−α/2^ for *f*_min_ < *f* < 10^−2^ Hz (Figure 4, red) with *f*_min_ < 10^−5^ Hz. Therefore, as explained in section 3.3.2 this model implies that the response of the neuron is affected by internal fluctuations over the scale of days (~ *f*^−1^_min_) or more, generating the *f*^−α^ behavior we observe in Figure 3. However, external input is remembered only for minutes (~ *f*^−1^_ext_).

Next, we examine two methods which allow us to probe *H*^ext^(*f*) directly and examine these predictions.

**Figure 5:**
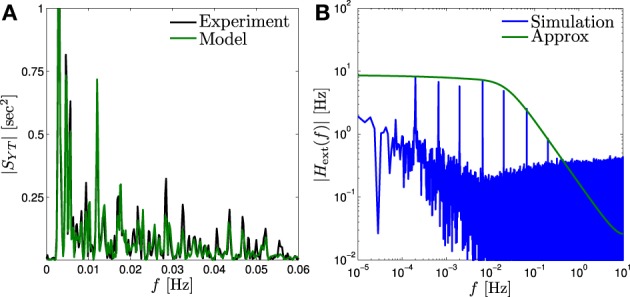
**Input memory in fitted model**. **(A)** Comparison of |*S*_*YT*_ (*f*)| of the fitted model (“Model”) to that estimated from the experimental confirms (“Experiment”) the prediction of the input filter |*H*^ext^(*f*)| for probed range. **(B)** This filter (“Approx”) can be probed more accurately by peaks of *Ŷ* (*f*) (“Simulation”), by applying a “sum of sines” input (Equation 43).

First, a simple method to probe the external input filter *H*^ext^(*f*) is through Equation (34). Allowing reliable estimation of *H*^ext^(*f*) in a certain frequency range requires a random process stimulation for which |*H*^ext^ (*f*) |^2^*S*_*T*_ (*f*) ≫ |*H*^int^(*f*)| in that range, as explained in Appendix B.2. To demonstrate this method we estimate *S*_*TY*_ (*f*) from the existing experimental data taken from Gal and Marom (2013), in which *S*_*T*_ (*f*) ~ *f*^−β^ (above some lower cutoff). In Figure 5A we compare this estimation with *S*_*TY*_ (*f*) in the HHMS model in a limited range where *S*_*T*_ (*f*) is sufficiently high for estimation to be accurate. It is similar to *S*_*TY*_ (*f*) from our fitted HHMS model, validating our estimate of *H*^ext^(*f*) for that frequency range.

Second, The filter *H*^ext^(*f*) could be probed more accurately and at lower frequencies – by sinusoidally modulating the input (the internal-stimulus intervals), analogously to the sinusoidally modulated input current used in Lundstrom et al. (2008, 2010) and Pozzorini et al. (2013),

(43) $${\hat{T}}_{m}=T_{\text{amp}}\sum_{l=1}^{L}\sin\left(2\pi f_{l}T_{*}m\right).$$

As we explain in Appendix B.3, in this case the output of the neuron would be

Y^m = ∑l = 1LTamp|Hext(fl)|sin(2πflT∗m+∠Hext(fl))+n"oise."

This allows us to easily estimate |*H*^ext^(*f*)| using the peaks of *T*_amp_^−1^ *Ŷ* (*f*) (the Fourier transform of *T*^−1^_amp_ *Ŷ*_*m*_) at frequencies *f*_*l*_, as we demonstrate in Figure 5B, using our fitted HHMS model.

#### 3.4.4. Additional predictions

As explained in Gal et al. (2010) and Soudry and Meir (2012) the latency of the AP can serve as an indicator of the cell's excitability. Specifically, this is true in the HHMS model, for periodical stimulus and p_*_ = 1, where the PSD of the latency, *S*_*L*_ (*f*), is a shifted and scaled version of *S*_*Y*_ (*f*) with p_*_ → 1 (see section 4.4.6 in Soudry and Meir, 2014). Therefore, in the HHMS model we also have *S*_*L*_ (*f*) ∝ *f*^−α^ approximately (neglecting logarithmic factors).

Next, suppose we vary some measurable stimulation parameter, such as the mean stimulation rate *T*^−1^_*_. How would this affect the shape of the filters we derived? The analytical results allow us to calculate this explicitly in the HHMS model.

First, we consider the gain of the external input filter *H*^ext^(*f*) (i.e., *H*^ext^(0)). As we explain in Appendix A.7, if *f* ≪ *f*_cutoff_, than

(44) $$H^{\text{ext}}\left(f\right)\approx p_{*}T_{*}^{-1}={\bar{f}}_{\text{out}},$$

which is the mean firing rate of the neuron – a simply measurable quantity.

Second, how would *H*_int_ (*f*) change if T_*_ is varied? Since *H*_int_ (*f*) is directly measurable only through *S*_*Y*_ (*f*) (Equation 36), we consider *S*_*Y*_ (*f*) instead. From Equation (59) it is clear that if *S*_*Y*_ (*f*) ~ *f*^−α^ approximately at low frequencies then the exponent α should not depend much on any external parameter (assuming 0 < p_*_ < 1). This was observed experimentally when the stimulation rate (*T*^−1^_*_) was varied, as can be seen in Figure 1G in Gal and Marom (2013).

## 4. Discussion

### 4.1. Generating *f*^−α^ PSD

In this work we aim to explain biophysically the phenomenon of *f*^−α^ behavior in the response of isolated neurons, and explore its implications on the input–output relation of the neuron. We do this under a regime of sparse stimulation (Figure 1), and in the biophysical framework of stochastic conductance-based models (CBMs, Equations 4–6). In this setting our analytical results (Soudry and Meir, 2014) can be used to derive a closed form expression for the Power Spectral Density (PSD, Equation 10) based on the parameters of the slow kinetics in the CBM. This PSD is affected by the closed-loop interaction – the slow dynamics affect the AP response, which, in turn, feeds back and affects the kinetics of the slow processes (section 2.3.2). Moreover, the contribution of each slow process to the PSD can be exactly quantified (section 2.3.3), as we demonstrate using a simple model (section 2.3.4).

These mathematical results expose the large parameter degeneracy of CBMs (Marder and Goaillard, 2006; Soudry and Meir, 2014), i.e., that many “different” models will quantitatively produce the same behavior. Due to the degeneracy of CBMs, we first aimed to derive rather general sufficient conditions for the generation of *f*^−α^ noise in a CBM (section 3.2.1). These conditions indicate which types of CBMs can generate the observed behavior. We show that, in order to generate *f*^−α^ behavior, neurons should have intrinsic fluctuations (e.g., due to ion channel noise), and have a number of slow processes with a large range of timescales, “covering” the entire range over which *f*^−α^ statistics is observed. Furthermore, the parameters of these processes must be scaled in a certain way in order to generate *f*^−α^ noise with a specific α (Equation 26).

We implement these constraints in a minimal CBM (section 3.2.2), in which the slow processes are uncoupled, except through the voltage, as in Soudry and Meir (2012). Initially, we find that the specific scaling relation can be achieved either by scaling the (1) conductances or (2) the ion channel numbers. This scaling implies that slower processes will be either (1) “stronger” or (2) “noisier.” However, the “feedback” effect in the model (the slow process being affected by the APs) prevents *f*^−α^ statistics from being generated in case (1). In contrast, option (2) can robustly generate the observed *f*^−α^ statistics in the neuronal response for any 0 < α < 2 (Equation 30 and Figure 6).

Naturally, outside of the framework of CBMs (Equations 4–6) long term correlations may be modeled differently, since there are numerous distinct ways to generate power law distributions (Newman, 2005). For example, as numerically demonstrated in Gal and Marom (2013), 1/f statistics in neuronal firing patterns can be generated by assuming global (cooperative) interactions between ion channels (i.e., not through the voltage). Biophysically, the significance of interactions between ion channels is still not clear (Naundorf et al., 2006 and Brief Communications arising), but other cellular processes that might affect excitability on slower timescales clearly exhibit interactions (e.g., gene regulation networks Davidson and Levin, 2005). Mathematically, such interactions render the slow dynamics (Equation 6) non-linear at constant voltage (Gillespie, 2000). It would be interesting to generalize the theory we presented here in order to understand how to tune the PSD in such a non-linear setting, since this has the potential to further reduce the number of parameters and model complexity.

### 4.2. Biophysical implementation

We examine our theoretical predictions numerically. We do this using a stochastic Hodgkin Huxley type model with slow sodium inactivation that was previously fitted to the basic experimental results (Soudry and Meir, 2012). We extend this model to include four additional slow processes, which resemble slow sodium inactivation (Appendix C.2). The only difference is that each process is slower than the previous one, and has a lower number of ion channels, according to the specific scaling relation that was derived. The resulting model indeed generates *f*^−α^ noise, and is demonstrated numerically (Figure 3) to fit the experimental results of Gal et al. (2010). This is the first time, to our knowledge, that a cortical neuron model (either biophysical or phenomenological) reproduces experimental results over such long timescales. Notably, without the analytical results, it would be hard to tune the parameters of a biophysical neuron, due to the large number of unknown parameters.

Previous works (Lowen et al., 1999; Soen and Braun, 2000) demonstrated numerically that, even with constant current stimulation, incorporating slow processes into an excitable cell model can generate *f*^−α^ in its response. In Lowen et al. (1999) a HH model was extended to include multiple slow processes with scaled rates in the potassium channel produced *f*^−α^ firing rate response. Their model produced an exponent of α ≈ 0.5, replicating experiments measurements from the auditory nerves. Another work (Soen and Braun, 2000), aiming to reproduce the activity of heart cells, produced long term correlations with α ≈ 1.6 − 2 using a reflected diffusion process.

The identity of the specific slow processes involved in generating *f*^−α^ remains a mystery at this point, since there are many possible mechanisms which can modulate the excitability of the cell in such long timescales. For example, ion channel numbers, conductances and kinetics are constantly being regulated and may change over time (e.g., Levitan, 1994; Staub et al., 1997). Also, the ionic concentrations in the cell depend on the activity of the ionic pumps, which can be affected by metabolism (Silver et al., 1997). Finally, the spike initiation region can significantly shift its location with time (e.g., 17 μm distally during 48 h of high activity Grubb and Burrone, 2010), and so can cellular neurites (Paola et al., 2006; Nishiyama et al., 2007). Only after such slow processes are quantitatively characterized, can we determine their effect on the neuron's excitability at long timescales.

### 4.3. The input–output relation

The linearized input–output relation of the fitted CBM was derived using the methods described in Soudry and Meir (2014). This linearized relation decomposes the contributions of external inputs and internal fluctuations to the response of the neuron. This decomposition (Equation 33) shows that even though the neuron can “remember” its intrinsic fluctuations over timescales of days, its memory of past pulse inputs can be limited to a shorter timescale of ~10^2^ s (Figure 4). Notably, the neuron has this limited memory for such inputs even though processes on much slower timescales exist in the model.

In the introduction we mentioned previous works (Lundstrom et al., 2008, 2010; Pozzorini et al., 2013) which also described the temporal integration in the neuron using power-law filters, although in a rather different (non-sparse) stimulation regime. Our fitted model indicates that similar power-law integration still occurs at very long timescales. However, it is not the input that is being integrated, but the internal fluctuations in the neuron, and this is what drives the *f*^−α^ statistics measured by Gal et al. (2010). Also, as in Lundstrom et al. (2008, 2010) and Pozzorini et al. (2013), the neuronal response in our model is indeed affected by the last 10 s of its external inputs. However, our model suggests the response will not be significantly affected by spike perturbations in its input that occurred more than 10^2^ s ago.

Qualitatively, this specific timescale of the input memory stems from the “fastest slow negative feedback process” in the model (in this specific model, slow sodium inactivation). This process responds to perturbations in the input which change the firing rate much more quickly than all the other slow processes. Its response to perturbation brings the firing rate back to its steady state, before slower processes even register that the firing rate has changed. Therefore, effectively, these slower processes do not store much information about input perturbations. We suggest experiments to test input memory directly, by using *f*^−α^ stimulation (Figure 5A), “sum of sines” stimulation (Figure 5B) and a variation of the mean stimulation rate (Equation 44 and Supplementary Figure 2).

### 4.4. Significance

This work makes several practical contributions. First, our results impose specific constraints on the slow processes that modulate the excitability on very long timescales (e.g., a ratio between timescales and channel numbers). Such constraints facilitate the construction of neuronal models with “realistic” input–output relations over extended timescales. Hopefully, these constraints may also help to identify the relevant slow biophysical processes. Second, our results suggest that for sparse spiking inputs, the memory of a cortical neuron stretches back to the last minute of its input, but not much more. This limit could be especially relevant when fitting statistical models to neuronal data, and for setting limitations on neuronal computations.

As for the functional significance, it is still not clear why the neuronal response fluctuates so wildly, especially at long timescales. We end this paper by offering some speculations on this issue. We see three possible scenarios. One possibility is that these fluctuations are beneficial. For example, such non-stationary fluctuations should increase network heterogeneity, which may be advantageous (Tessone et al., 2006; Padmanabhan and Urban, 2010). Another possibility is that these fluctuations do not affect neural response when the neuron is connected within a network. For example, this could be due to network feedback canceling slow changes in excitability. Finally, it is possible that such slow fluctuations are deleterious, an unavoidable “noise” generated by the non-stationary environment of the cell. Interestingly, *f*^−α^ noise imposes important constraints on electronic circuits, and was predicted to impose similar constraints on neural circuits (Sarpeshkar, 1998).

### Conflict of interest statement

The authors declare that the research was conducted in the absence of any commercial or financial relationships that could be construed as a potential conflict of interest.

## Footnotes

^1^I.e., if ∀*t* : *I*(*t*) = 0, then the probability that a neuron will fire is negligible – on any relevant finite time interval (e.g., minutes or days).

^2^A semi-analytic derivation is an analytic derivation in which some terms are obtained by relatively simple numerics.

^3^For example, this can happen if the kinetic rates all have low voltage threshold, resulting in **A**_+_ ≈ **A**_ and **b**_+_ ≈ **b**_.

^4^I.e., in all neurons for which 0 < *p*_*_ < 1.

^5^Otherwise, 0.25 ≥ *p*_*_ − *p*_*_^2^ = 〈 *Ŷ*_*m*_^2^ 〉 ≥ 2 $\int_{0}^{f_{\max}}S_{Y}$ (*f*)*df* = ∞, which is a contradiction.

^6^Also simulations take a long time, since experiments, as in Gal et al. (2010), are days-long.

^7^E.g., in Equation (16) many different parameters would give the same *c*_*k*_.

^8^Near the edges, **w** → 0 (Equation 80 in Soudry and Meir, 2014), and so κ (*f*) → 1.

^9^I.e., Equations (4–6), with the same assumptions as we had in section 2.3.1.
