# Supplementary material for: The neuronal response at extended timescales: long-term correlations without long-term memory
Source: Front Comput Neurosci. 2014 Apr 1;8:35. doi: 10.3389/fncom.2014.00035 (PMC3978321; doi:10.3389/fncom.2014.00035)
Supplement: Supplementary file 1 [file Presentation1.PDF]

Appendix

A Detailed derivations

A.1 Derivation of Eqs. 15-16

Recall Eq. 13.

$$S_Y^o(f) \triangleq T_* \sigma_e^2 + \mathbf{w}^\top \mathbf{H}_o(-f)\,\mathbf{D}_* \mathbf{H}_o^\top(f)\,\mathbf{w}\,.$$

Suppose that $\mathbf{A}_*$ is diagonalizable, so that we can write $\mathbf{A}_* = \mathbf{U}\tilde{\mathbf{A}}\mathbf{V}$, where $\mathbf{U} = \mathbf{V}^{-1}$ and $\tilde{\mathbf{A}}$ is some diagonal matrix. In that case

$$\begin{aligned}
& \mathbf{w}^\top \mathbf{H}_o(-f)\,\mathbf{D}_* \mathbf{H}_o^\top(f)\,\mathbf{w} \\
= \; & \mathbf{w}^\top \left(-2\pi f i \mathbf{I} - \mathbf{A}_*\right)^{-1} \mathbf{D}_* \left(2\pi f i \mathbf{I} - \mathbf{A}_*^\top\right)^{-1} \mathbf{w} \\
= \; & \mathbf{w}^\top \left(-2\pi f i \mathbf{I} - \mathbf{U}\tilde{\mathbf{A}}\mathbf{V}\right)^{-1} \mathbf{D}_* \left(2\pi f i \mathbf{I} - \mathbf{V}^\top \tilde{\mathbf{A}} \mathbf{U}^\top\right)^{-1} \mathbf{w} \\
= \; & \mathbf{w}^\top \mathbf{V}^{-1} \left(-2\pi f i \mathbf{I} - \tilde{\mathbf{A}}\right)^{-1} \mathbf{U}^{-1} \mathbf{D}_* \left(\mathbf{U}^{-1}\right)^\top \left(2\pi f i \mathbf{I} - \tilde{\mathbf{A}}\right)^{-1} \left(\mathbf{V}^{-1}\right)^\top \mathbf{w} \\
= \; & \tilde{\mathbf{w}}^\top \left(-2\pi f i \mathbf{I} - \tilde{\mathbf{A}}\right)^{-1} \tilde{\mathbf{D}} \left(2\pi f i \mathbf{I} - \tilde{\mathbf{A}}\right)^{-1} \tilde{\mathbf{w}}
\end{aligned}$$

where in the last line we denoted $\tilde{\mathbf{w}} = \left(\mathbf{V}^{-1}\right)^\top \mathbf{w}$ and $\tilde{\mathbf{D}} = \mathbf{U}^{-1}\mathbf{D}_* \left(\mathbf{U}^{-1}\right)^\top$. Denoting $D_{kj} = \left(\tilde{\mathbf{D}}\right)_{kj}$, $A_{kj} = \left(\tilde{\mathbf{A}}\right)_{kj}$, $A_k = A_{kk}$ and $w_k = (\tilde{\mathbf{w}})_k$, and noting that $\tilde{\mathbf{D}}$ is a symmetric matrix (since $\mathbf{D}_*$ is symmetric), the last line can be decomposed into partial fractions in the following way

$$\begin{aligned}
& \tilde{\mathbf{w}}^\top \left(-2\pi f i \mathbf{I} - \tilde{\mathbf{A}}\right)^{-1} \tilde{\mathbf{D}} \left(2\pi f i \mathbf{I} - \tilde{\mathbf{A}}\right)^{-1} \tilde{\mathbf{w}} \\
= \; & \sum_{k,j} \frac{w_k D_{kj} w_j}{\left(-2\pi f i - A_k\right)\left(2\pi f i - A_j\right)} \\
= \; & -\sum_{k,j} \frac{w_k D_{kj} w_j}{A_k + A_j} \left[\frac{1}{-2\pi f i - A_k} + \frac{1}{2\pi f i - A_j}\right] \\
= \; & \sum_{k,j} \frac{w_k D_{kj} w_j}{A_k + A_j} \left[\frac{1}{2\pi f i + A_k} + \frac{1}{-2\pi f i + A_k}\right] \\
= \; & 2\sum_{k,j} \frac{w_k D_{kj} w_j}{A_k + A_j} \left[\frac{A_k}{\left(2\pi f\right)^2 + A_k^2}\right] \\
= \; & \sum_k \left(\sum_j w_k D_{kj} w_j \frac{2A_k}{A_k + A_j}\right) \frac{1}{\left(2\pi f\right)^2 + A_k^2}
\end{aligned}$$

which gives Eqs. 15-16.

A.2 Complex poles

Suppose the partial fraction decomposition of $S_Y^o(f)$ (Eq. 15) contains complex poles. Since $S_Y^o(f)$ is real, these poles must appear for complex conjugate pairs λ, $\bar{\lambda}$, with complex conjugate amplitudes, as follows

$$\begin{aligned} & \frac{c}{(2\pi f)^2+\lambda^2}+\frac{\bar{c}}{(2\pi f)^2+\bar{\lambda}^2} \\ = & \frac{c\left((2\pi f)^2+\bar{\lambda}^2\right)+\bar{c}\left((2\pi f)^2+\lambda^2\right)}{\left((2\pi f)^2+\lambda^2\right)\left((2\pi f)^2+\bar{\lambda}^2\right)} \\ = & \frac{(2\pi f)^2(c+\bar{c})+c\bar{\lambda}^2+\bar{c}\lambda^2}{(2\pi f)^4+(2\pi f)^2\left(\lambda^2+\bar{\lambda}^2\right)+|\lambda|^2}\,. \end{aligned} \tag{45}$$

Denoting $e^{i\theta}\triangleq\lambda/|\lambda|$ and $e^{i\phi}\triangleq c/|c|$ we can write Eq. 45 as

$$\begin{aligned} & 2|c|\frac{(2\pi f)^2\cos(\phi)+|\lambda|^2\cos(\phi+2\theta)}{(2\pi f)^4+2(2\pi f)^2|\lambda|^2\cos(2\theta)+|\lambda|^4} \\ = & \begin{cases} \frac{2|c|\cos(\phi)}{(2\pi f)^2} & \text{, if } 2\pi f\gg|\lambda| \\ \frac{|c|}{|\lambda|^2}\frac{\cos(\phi)+\cos(\phi+2\theta)}{1+\cos(2\theta)} & \text{, if } 2\pi f\sim|\lambda| \\ \frac{2|c|\cos(\phi+2\theta)}{|\lambda|^2} & \text{, if } 2\pi f\ll|\lambda| \end{cases}\,. \end{aligned} \tag{46}$$

Note that for $2\pi f\sim|\lambda|$, this can go to infinity if $\theta\rightarrow\pm\pi/2$. In comparison, for a real pole

$$\frac{c}{(2\pi f)^2+\lambda^2}=\begin{cases} \frac{c}{(2\pi f)^2} & \text{, if } 2\pi f\gg|\lambda| \\ \frac{c}{2|\lambda|^2} & \text{, if } 2\pi f\sim|\lambda| \\ \frac{c}{|\lambda|^2} & \text{, if } 2\pi f\ll|\lambda| \end{cases}\,.$$

Therefore, the asymptotic behavior is similar, except that for a simple pole there is a different pre-factor in each region.

A.3 Derivation of Eq. 26

From Eq. 24, we have

$$S_Y^o(f) = \int\frac{\rho(\lambda)\,d\lambda}{(2\pi f)^2+\lambda^2}\,.$$

We define $\tilde{\rho}(\lambda) = |\lambda|^{\alpha-1}\rho(\lambda)$ and recall that $\rho(\lambda) = 0$ outside of the range $|\lambda_M| < |\lambda| < |\lambda_{L+1}|$. We obtain

$$\begin{aligned} S_Y^o(f) &= \int_{\lambda_{L+1}}^{\lambda_M} \frac{(-\lambda)^{1-\alpha}\tilde{\rho}(\lambda)}{(2\pi f)^2 + \lambda^2} d\lambda \\ &\overset{u=\frac{-\lambda}{2\pi f}}{=} (2\pi f)^{-\alpha} \int_{-\lambda_M/2\pi f}^{-\lambda_{L+1}/2\pi f} \frac{u^{1-\alpha}\tilde{\rho}(-2\pi f u)}{1+u^2} du \\ &\rightarrow (2\pi f)^{-\alpha} \int_0^{\infty} \frac{u^{1-\alpha}\tilde{\rho}(-2\pi f u)}{1+u^2} du \end{aligned} \tag{47}$$

where we assumed in the last line that$|\lambda_{L+1}| \gg 2\pi f \gg |\lambda_M|$ and recall that $0 < \alpha < 2$ (note that if $\alpha < 0$ or $\alpha > 2$, the last integral will diverge). Therefore, clearly

$$S_Y^o(f) \propto f^{-\alpha} \tag{48}$$

in that range if $\tilde{\rho}(\lambda)$ is constant.

However, is this also necessary condition? In order that Eq. 48 would remain true we must have

$$\int_0^{\infty} \frac{u^{1-\alpha}\tilde{\rho}(-2\pi f u)}{1+u^2} du = C, \tag{49}$$

where C is some finite constant. To show that this must imply that $\tilde{\rho}(\lambda)$ is constant we define $x = -\ln(2\pi f)$, and $\hat{\rho}(-\ln(-x)) \triangleq \tilde{\rho}(x)$. Eq. 49 then becomes

$$\int_0^{\infty} \frac{u^{1-\alpha}\hat{\rho}(x - \ln u)}{1+u^2} du = C\,.$$

Changing variables to $v = \ln u$ (so $u = e^v$ and $du = e^v dv$) we obtain

$$\int_{-\infty}^{\infty} \frac{e^{-\alpha v}\hat{\rho}(x-v)}{1+e^{-2v}} dv = (\psi * \hat{\rho})(x) = C \tag{50}$$

with $*$ denoting the convolution operation and

$$\psi(x) \triangleq \frac{e^{-\alpha v}}{1+e^{-2v}}\,.$$

Assuming that the (generalized) Fourier transform of ρ,

$$\varrho(\omega) \triangleq \int_{-\infty}^{\infty} \hat{\rho}(x) e^{-i\omega x} dx\,,$$

exists, we take the Fourier transform of Eq. 50, and obtain

$$\Psi(\omega)\varrho(\omega) = 2\pi C\delta(\omega) \tag{51}$$

where $\delta(\omega)$ is Dirac's delta function, and

$$\Psi(\omega) \triangleq \int_{-\infty}^{\infty} \psi(x) e^{-i\omega x} dx = \frac{\pi}{2\sin(\pi(\alpha + i\omega)/2)}\,. \tag{52}$$

Therefore, From Eqs. 51-52, we obtain

$$\varrho(\omega) = \frac{C\delta(\omega)}{\Psi(0)} = 4C\sin(\pi\alpha/2)\,\delta(\omega)$$

and so

$$\hat{\rho}(x) = 2\pi^{-1}C\sin(\pi\alpha/2),$$

a constant. Therefore, $\tilde{\rho}(x)$ is also a constant, which is what we wanted to prove.

To summarize, we find that

$$S_Y^o(f) = Cf^{-\alpha}. \tag{53}$$

if and only if

$$\rho(\lambda) = 2\pi^{-1}C\sin(\pi\alpha/2)\,|\lambda|^{1-\alpha}. \tag{54}$$

A.4 Derivation of Eq. 30 and related matters

In the diagonal model presented in section 3.2.2

$$S_Y^o(f) = T_*\sigma_e^2 + \sum_{k=1}^{M} \frac{c_k}{(2\pi f)^2 + \lambda_k^2}, \tag{55}$$

with $\lambda_k = \lambda_1\epsilon^{k-1}$, and

$$c_k = \frac{w_k^2}{N_{s,k}} \frac{\gamma_{*,k}\delta_{*,k}}{\gamma_{*,k}+\delta_{*,k}} \tag{56}$$

where $w_k = w_1\epsilon^{-\mu(k-1)}$ and $N_{s,k} = N_{s,1}\epsilon^{\nu(k-1)}$. Therefore $c_k = c_1\epsilon^{(1-\eta)(k-1)}$ with $\eta = \nu + 2\mu$, and we can write

$$S_Y^o(f) = T_*\sigma_e^2 + c_1 \sum_{j=0}^{M-1} \frac{\epsilon^{j(1-\eta)}}{(2\pi f)^2 + \lambda_1^2\epsilon^{2j}}.$$

In section A.5, we find that for $|\lambda_M| \ll 2\pi f \ll |\lambda_1|$

$$\sum_{j=0}^{M-1} \frac{\epsilon^{j(1-\eta)}}{(2\pi f)^2 + \lambda_1^2\epsilon^{2j}} \approx g(\epsilon,\eta) \times \begin{cases} \epsilon^{M(1-\eta)}(2\pi f)^{-2} & \text{, if } \eta > 1 \\ \ln\left(|\lambda_1|\epsilon^M/(2\pi f)\right)(2\pi f)^{-2} & \text{, if } \eta = 1 \\ \lambda_1^{-2}\left(\frac{2\pi f}{|\lambda_1|}\right)^{-(1+\eta)} & \text{, if } 1 > \eta > -1 \\ \lambda_1^{-2}\ln(-2\pi f/\lambda_1) & \text{, if } \eta = -1 \\ \lambda_1^{-2} & \text{, if } \eta < -1 \end{cases} \tag{57}$$

where $g(\epsilon,\eta)$ is some proportionality constant that depends only on ϵ and η. This approximation immediately gives us $S_Y^o(f)$. It is accurate both in the limit that the poles are sparse ($\epsilon \to 0$) and in the limit that the poles are dense ($\epsilon \to 1^-$).

Next, we note that $a_k = a_1\epsilon^{k-1}$ with $a_1 = \tau_{\mathrm{AP}}\left(\gamma_{*,1}(\delta_{+,1}-\delta_{-,1}) - (\gamma_{+,1}-\gamma_{-,1})\delta_{*,1}\right)/(\gamma_{*,1}+\delta_{*,1})$. Applying these substitutions to Eq. 17, we obtain

$$\kappa(f) = 1 - T_*^{-1} w_1 a_1 \sum_{j=0}^{M-1} \frac{\epsilon^{(1-\mu)j}}{2\pi f i - |\lambda_1| \epsilon^j}$$
$$= 1 + T_*^{-1} w_1 a_1 \sum_{j=0}^{M-1} \frac{\epsilon^{(1-\mu)j} \left(2\pi f i + \lambda_1 \epsilon^j\right)}{(2\pi f)^2 + (\lambda_1 \epsilon^j)^2}.$$

Therefore

$$\frac{\kappa(f) - 1}{T_*^{-1} w_1 a_1 \lambda_1} = \frac{2\pi f}{\lambda_1} i \cdot g(\epsilon, \mu) \times \begin{cases} \epsilon^{M(1-\mu)} (2\pi f)^{-2} & ,\text{if } \mu > 1 \\ \ln\left(|\lambda_1| \epsilon^M / (2\pi f)\right) (2\pi f)^{-2} & ,\text{if } \mu = 1 \\ \lambda_1^{-2} \left(\frac{2\pi f}{|\lambda_1|}\right)^{-(1+\mu)} & ,\text{if } 1 > \mu > -1 \\ \lambda_1^{-2} \ln(2\pi f / |\lambda_1|) & ,\text{if } \mu = -1 \\ \lambda_1^{-2} & ,\text{if } \mu < -1 \end{cases}$$
$$+ \; g(\epsilon, \mu - 1) \times \begin{cases} \epsilon^{M(2-\mu)} (2\pi f)^{-2} & ,\text{if } \mu > 2 \\ \ln\left(|\lambda_1| \epsilon^M / (2\pi f)\right) (2\pi f)^{-2} & ,\text{if } \mu = 2 \\ \lambda_1^{-2} \left(\frac{2\pi f}{|\lambda_1|}\right)^{-\mu} & ,\text{if } 2 > \mu > 0 \\ \lambda_1^{-2} \ln(2\pi f / |\lambda_1|) & ,\text{if } \mu = 0 \\ \lambda_1^{-2} & ,\text{if } \mu < 0 \end{cases}$$
$$= \begin{cases} g(\epsilon, \mu - 1) \epsilon^{M(2-\mu)} \left(\frac{2\pi f}{|\lambda_1|}\right)^{-2} & ,\text{if } \mu > 2 \\ g(\epsilon, \mu - 1) \ln\left(|\lambda_1| \epsilon^M / (2\pi f)\right) \left(\frac{2\pi f}{|\lambda_1|}\right)^{-2} & ,\text{if } \mu = 2 \\ g(\epsilon, \mu - 1) \left(\frac{2\pi f}{|\lambda_1|}\right)^{-\mu} & ,\text{if } 2 > \mu \geq 1 \\ (i g(\epsilon, \mu) + g(\epsilon, \mu - 1)) \left(\frac{2\pi f}{|\lambda_1|}\right)^{-\mu} & ,\text{if } 1 > \mu > 0 \\ g(\epsilon, \mu - 1) \ln(2\pi f / |\lambda_1|) & ,\text{if } \mu = 0 \\ g(\epsilon, \mu - 1) & ,\text{if } \mu < 0 \end{cases}$$

where we used the fact that $2\pi f \ll |\lambda_1|$. Now, if the constant 1 is negligible, then we have

$$|\kappa(f)|^{-2} \approx T_*^2 w_1^{-2} a_1^{-2} \lambda_1^2 \times \begin{cases} g^{-2}(\epsilon, \mu - 1) \epsilon^{-2M(2-\mu)} \left(\frac{2\pi f}{|\lambda_1|}\right)^4 & ,\text{if } \mu > 2 \\ g^{-2}(\epsilon, \mu - 1) \left(\ln\left(|\lambda_1| \epsilon^M / (2\pi f)\right)\right)^{-2} \left(\frac{2\pi f}{|\lambda_1|}\right)^4 & ,\text{if } \mu = 2 \\ g^{-2}(\epsilon, \mu - 1) \left(\frac{2\pi f}{|\lambda_1|}\right)^{2\mu} & ,\text{if } 2 > \mu \geq 1 \\ \left(g^2(\epsilon, \mu) + g^2(\epsilon, \mu - 1)\right)^{-1} \left(\frac{2\pi f}{|\lambda_1|}\right)^{2\mu} & ,\text{if } 1 > \mu > 0 \\ g^{-2}(\epsilon, \mu - 1) \left(\ln(2\pi f / |\lambda_1|)\right)^{-2} & ,\text{if } \mu = 0 \\ g^{-2}(\epsilon, \mu - 1) & ,\text{if } \mu < 0 \end{cases} \quad (58)$$

Therefore, since $S_Y(f) = S_Y^o(f) |\kappa(f)|^{-2}$, we have (assuming $\sigma_e^2 T_*$ is negligible), for

$\mu = 0$

$$S_Y(f) \approx \frac{g^{-2}(\epsilon,-1)\, g(\epsilon,\eta)\, T_*^2 c_1}{w_1^2 a_1^2 \ln^2(2\pi f/|\lambda_1|)} \times \begin{cases} \epsilon^{M(1-\eta)} \left(\frac{2\pi f}{|\lambda_1|}\right)^{-2} & ,\text{if } \eta > 1 \\ \ln\left(|\lambda_1|\,\epsilon^M/(2\pi f)\right) \left(\frac{2\pi f}{|\lambda_1|}\right)^{-2} & ,\text{if } \eta = 1 \\ \left(\frac{2\pi f}{|\lambda_1|}\right)^{-(1+\eta)} & ,\text{if } 1 > \eta > -1 \\ \ln\left(\frac{2\pi f}{|\lambda_1|}\right) & ,\text{if } \eta = -1 \\ 1 & ,\text{if } \eta < -1 \end{cases}, \tag{59}$$

This approximation captures well the asymptotic behavior of $S_Y(f)$ (Eq. 10) for the HHMS model with $|\lambda_M| \ll 2\pi f \ll |\lambda_1|$, as can be seen in Fig. 6 for various values of η. Note that from Eq. 56 $c_1 \propto w_1^2/N_{s,1}$. Therefore: (1) the parameter w_1 has effectively been canceled out from the expression and does not affect $S_Y(f)$ in this range, (2) $S_Y(f) \propto N_{s,1}^{-1}$.

For $\mu > 0$, we recall that $\eta = \nu + 2\mu$, and note that in the ("relevant") range $-1 < \eta < 1$, due to the effects of feedback (Eq. 58),

$$S_Y(f) = S_Y^o(f)\,|\kappa(f)|^{-2} \propto \begin{cases} f^{-(1+\nu+2\mu)} & ,\mu < 0 \\ f^{-(1+\nu)} & ,2 > \mu > 0 \\ f^{-(1+\nu+2\mu-4)} & ,\mu > 2 \end{cases}$$

so the contribution of μ, the scaling in $\mathbf{w}$, can either decrease the exponent of $S_Y(f)$ (if $\mu < 0$), not change it (if $2 > \mu > 0$) or increase it only if ν is already quite negative ($\nu < -2$). Therefore, ν does not really "help" in increasing the exponent of $S_Y(f)$. If, for example, $\nu = 0$, then we can have $S_Y(f) \propto f^{-\alpha}$ with $\alpha \leq 1$ (which is lower than the observed value of $\alpha \approx 1.4$). Therefore, the "simplest" choice to generate $S_Y(f) \propto f^{-\alpha}$ would be $\mu = 0$ and $\nu = \eta$ with η slightly higher than $\alpha - 1$ (due to the logarithmic correction in Eq. 59).

A.5 Derivation of Eq. 57

We wish to calculate the sum

$$\sum_{j=0}^{M-1} \frac{\epsilon^{j(1-\eta)}}{(2\pi f)^2 + \lambda_1^2 \epsilon^{2j}}.$$

assuming that

$$|\lambda_1|\,\epsilon^{M-1} \ll 2\pi f \ll |\lambda_1|. \tag{60}$$

We shall do this in two opposing limits - when the poles are sparse, and when they are dense.

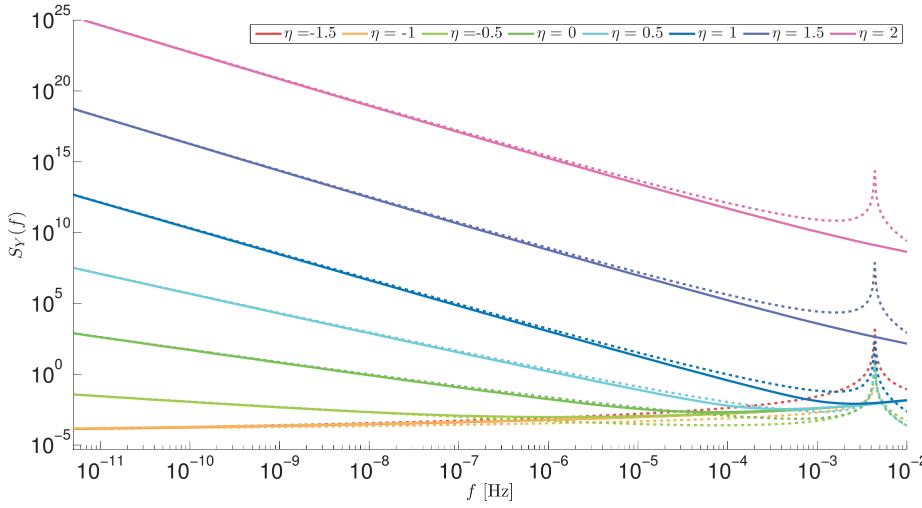

Figure 6: Analytic expression $S_Y(f)$ (full line, Eq. 10) for the HHMS model and its asymptotic approximation (dotted line, Eq. 59, with fitted pre-factors), for different values of η, with $I = 7.5\mu A$, $M = 20$ and $\epsilon = 0.2$. Note that the slopes match the analytical approximation well and increase with η when $\eta \in [-1, 1]$. However, near $f \sim f_{\max}$ the asymptotic approximation becomes inaccurate (and even diverges, due to the logarithmic factor).

A.5.1 Sparse poles

First, we assume the poles are sparse (*i.e.*, well separated, so $\epsilon \ll 1$). We denote $j_* \triangleq \lceil \ln(2\pi f / |\lambda_1|) / \ln \epsilon \rceil$ (where $\lceil \cdot \rceil$ denotes the upper integer values), so

$$
\begin{aligned}
\sum_{j=0}^{M-1} \frac{\epsilon^{j(1-\eta)}}{(2\pi f)^2 + \lambda_1^2 \epsilon^{2j}} &\approx \sum_{j=j_*}^{M-1} \frac{\epsilon^{j(1-\eta)}}{(2\pi f)^2} + \sum_{j=0}^{j_*-1} \frac{\epsilon^{j(1-\eta)}}{\lambda_1^2 \epsilon^{2j}} \\
&= (2\pi f)^{-2} \sum_{j=j_*}^{M-1} \epsilon^{j(1-\eta)} + \lambda_1^{-2} \sum_{j=0}^{j_*-1} \epsilon^{-j(1+\eta)} \\
&\overset{\forall \eta \neq \pm 1}{=} (2\pi f)^{-2} \frac{\epsilon^{j_*(1-\eta)} - \epsilon^{M(1-\eta)}}{1 - \epsilon^{(1-\eta)}} + \lambda_1^{-2} \frac{1 - \epsilon^{-j_*(1+\eta)}}{1 - \epsilon^{-(1+\eta)}} \\
&\approx (2\pi f)^{-2} \frac{(-2\pi f / |\lambda_1|)^{(1-\eta)} - \epsilon^{M(1-\eta)}}{1 - \epsilon^{(1-\eta)}} + \lambda_1^{-2} \frac{1 - (2\pi f / |\lambda_1|)^{-(1+\eta)}}{1 - \epsilon^{-(1+\eta)}} \\
&\approx \begin{cases} (2\pi f)^{-2} \frac{\epsilon^{M(1-\eta)}}{\epsilon^{(1-\eta)} - 1} & , \text{if } \eta > 1 \\ \lambda_1^{-2} \left(\frac{2\pi}{|\lambda_1|} \right)^{-(1+\eta)} \left(\frac{1}{1-\epsilon^{(1-\eta)}} - \frac{1}{1-\epsilon^{-(1+\eta)}} \right) \cdot f^{-(1+\eta)} & , \text{if } 1 > \eta > -1 \\ \frac{\lambda_1^{-2}}{1-\epsilon^{-(1+\eta)}} & , \text{if } \eta < -1 \end{cases}
\end{aligned}
$$

where in the last line we used the assumption in Eq. 60. Next, for $\eta = 1$,

$$\begin{aligned}
\sum_{j=0}^{M-1} \frac{1}{(2\pi f)^2 + \lambda_1^2 \epsilon^{2j}} &\approx (2\pi f)^{-2} \sum_{j=j_*}^{M-1} 1 + \lambda_1^{-2} \sum_{j=0}^{j_*-1} \epsilon^{-2j} \\
&= (2\pi f)^{-2} (M - j_*) + \frac{\lambda_1^{-2} - (2\pi f)^{-2}}{1 - \epsilon^{-2}} \\
&\approx (2\pi f)^{-2} \ln \left(|\lambda_1| \epsilon^M / (2\pi f) \right) / \ln \epsilon \,,
\end{aligned}$$

where in the last line we used the assumption in Eq. 60. Next, for $\eta = -1$,

$$\begin{aligned}
\sum_{j=0}^{M-1} \frac{\epsilon^{2j}}{(2\pi f)^2 + \lambda_1^2 \epsilon^{2j}} &\approx (2\pi f)^{-2} \sum_{j=j_*}^{M-1} \epsilon^{-2j} + \lambda_1^{-2} \sum_{j=0}^{j_*-1} 1 \\
&= (2\pi f)^{-2} \frac{(2\pi f / |\lambda_1|)^2 - \epsilon^{2M}}{1 - \epsilon^2} + \lambda_1^{-2} j_* \\
&\approx \lambda_1^{-2} \ln (2\pi f / |\lambda_1|) / \ln \epsilon
\end{aligned}$$

A.5.2 Dense poles

Next, we assume the poles are dense (*i.e.*, very close to each other, so $1 - \epsilon \ll 1$). In this case, we denote, $\lambda_j = |\lambda_1| \epsilon^j$, $d\lambda_j = \lambda_{j-1} - \lambda_j = (1 - \epsilon) |\lambda_1| \epsilon^{j-1} = \left(\epsilon^{-1} - 1 \right) \lambda_j$, so

$$
\begin{aligned}
&\left(|\lambda_1|\right)^{1-\eta}\left(\epsilon^{-1}-1\right)\sum_{j=0}^{M-1}\frac{\epsilon^{j(1-\eta)}}{\left(2\pi f\right)^2+\lambda_1^2\epsilon^{2j}}\\
=&\sum_{j=0}^{M-1}\frac{\lambda_j^{-\eta}d\lambda_j}{\left(2\pi f\right)^2+\lambda_j^2}\\
\approx&\int_{|\lambda_1|\epsilon^{M-1}}^{|\lambda_1|}\frac{\lambda^{-\eta}d\lambda}{\left(2\pi f\right)^2+\lambda^2}\\
=&\left(2\pi f\right)^{-\eta-1}\int_{|\lambda_1|\epsilon^{M-1}}^{|\lambda_1|}\frac{\left(\lambda/2\pi f\right)^{-\eta}d\left(\lambda/2\pi f\right)}{1+\left(\lambda/2\pi f\right)^2}\\
=&\left(2\pi f\right)^{-\eta-1}\int_{|\lambda_1|\epsilon^{M-1}/2\pi f}^{|\lambda_1|/2\pi f}\frac{x^{-\eta}dx}{1+x^2}\\
\approx&\left(2\pi f\right)^{-\eta-1}\cdot\begin{cases}\int_{|\lambda_1|\epsilon^{M-1}/2\pi f}^{\infty}\frac{x^{-\eta}dx}{1+x^2} & \text{, if } \eta\geq 1\\ \int_0^{\infty}\frac{x^{-\eta}dx}{1+x^2} & \text{, if } 1>\eta>-1\\ \int_0^{|\lambda_1|/2\pi f}\frac{x^{-\eta}dx}{1+x^2} & \text{, if } \eta\leq -1\end{cases}\\
\approx&\left(2\pi f\right)^{-\eta-1}\cdot\begin{cases}\left(|\lambda_1|\epsilon^{M-1}/2\pi f\right)^{-\eta+1}/\left(\eta-1\right) & \text{, if } \eta>1\\ \ln\left(2\pi f/|\lambda_1|\epsilon^{M-1}\right) & \text{, if } \eta=1\\ \int_0^{\infty}\frac{x^{-\eta}dx}{1+x^2} & \text{, if } 1>\eta>-1\\ \ln\left(|\lambda_1|/2\pi f\right) & \text{, if } \eta=-1\\ \left(|\lambda_1|/2\pi f\right)^{-\eta-1}/\left(-\eta-1\right) & \text{, if } \eta<-1\end{cases}\\
=&\begin{cases}\frac{1}{\eta-1}\left(|\lambda_1|\epsilon^{M-1}\right)^{-\eta+1}\cdot\left(2\pi f\right)^{-2} & \text{, if } \eta>1\\ \ln\left(2\pi f/|\lambda_1|\epsilon^{M-1}\right)\cdot\left(2\pi f\right)^{-2} & \text{, if } \eta=1\\ \frac{\pi}{2\cos(\pi\eta/2)}\cdot\left(2\pi f\right)^{-\eta-1} & \text{, if } 1>\eta>-1\\ \ln\left(|\lambda_1|/2\pi f\right) & \text{, if } \eta=-1\\ \frac{1}{-\eta-1}|\lambda_1|^{-\eta-1} & \text{, if } \eta<-1\end{cases}.
\end{aligned}
$$

A.5.3 Summary

In the dense limit

$$
\sum_{j=0}^{M-1}\frac{\epsilon^{j(1-\eta)}}{\left(2\pi f\right)^2+\lambda_1^2\epsilon^{2j}}\approx\left(\epsilon^{-1}-1\right)^{-1}\begin{cases}\frac{1}{\eta-1}\left(\epsilon^{M-1}\right)^{-\eta+1}\cdot\left(2\pi f\right)^{-2} & \text{, if } \eta>1\\ \ln\left(2\pi f/|\lambda_1|\epsilon^{M-1}\right)\cdot\left(2\pi f\right)^{-2} & \text{, if } \eta=1\\ \frac{\pi\lambda_1^{-2}}{2\cos(\pi\eta/2)}\cdot\left(\frac{2\pi f}{|\lambda_1|}\right)^{-(\eta+1)} & \text{, if } 1>\eta>-1\\ \lambda_1^{-2}\ln\left(|\lambda_1|/2\pi f\right) & \text{, if } \eta=-1\\ \frac{1}{-\eta-1}\lambda_1^{-2} & \text{, if } \eta<-1\end{cases}.
$$

In the sparse pole limit

$$\sum_{j=0}^{M-1}\frac{\epsilon^{j(1-\eta)}}{(2\pi f)^2+\lambda_1^2\epsilon^{2j}} \quad\approx\quad \begin{cases} \frac{\epsilon^{M(1-\eta)}}{\epsilon^{(1-\eta)}-1}(2\pi f)^{-2} & \text{, if } \eta>1 \\ \left[\ln\left(|\lambda_1|\epsilon^M/(2\pi f)\right)/\ln\epsilon\right](2\pi f)^{-2} & \text{, if } \eta=1 \\ \lambda_1^{-2}\left(\frac{1}{1-\epsilon^{(1-\eta)}}-\frac{1}{1-\epsilon^{-(1+\eta)}}\right)\cdot\left(\frac{2\pi f}{|\lambda_1|}\right)^{-(1+\eta)} & \text{, if } 1>\eta>-1 \\ \lambda_1^{-2}\ln(2\pi f/|\lambda_1|)/\ln\epsilon & \text{, if } \eta=-1 \\ \frac{\lambda_1^{-2}}{1-\epsilon^{-(1+\eta)}} & \text{, if } \eta<-1 \end{cases}$$

So, in general, assuming some continuity between both limits

$$\sum_{j=0}^{M-1}\frac{\epsilon^{j(1-\eta)}}{(2\pi f)^2+\lambda_1^2\epsilon^{2j}}\approx c(\epsilon,\eta)\cdot\begin{cases} \epsilon^{M(1-\eta)}(2\pi f)^{-2} & \text{, if } \eta>1 \\ \ln\left(|\lambda_1|\epsilon^M/(2\pi f)\right)(2\pi f)^{-2} & \text{, if } \eta=1 \\ \lambda_1^{-2}\left(\frac{2\pi f}{|\lambda_1|}\right)^{-(1+\eta)} & \text{, if } 1>\eta>-1 \\ \lambda_1^{-2}\ln(2\pi f/|\lambda_1|) & \text{, if } \eta=-1 \\ \lambda_1^{-2} & \text{, if } \eta<-1 \end{cases} \tag{61}$$

where $c(\epsilon,\eta)$ is some proportionality constant that depends only on ϵ and η. Note that in both cases $c(\epsilon,\eta)$ diverges when $\eta\to\pm1,\infty$ or when $\epsilon\to1$.

A.6 Derivation of Eq. 41

From Eq. 37, using similar notation and analysis as in the previous section, we write

$$H^{\text{ext}}(f)=H_o^{\text{ext}}(f)/\kappa(f)$$

with

$$H_o^{\text{ext}}(f)\quad\triangleq\quad T_*^{-1}\mathbf{w}^\top(2\pi fi\mathbf{I}-\mathbf{A}_*)^{-1}\mathbf{d}$$

being the "open loop" version of $H_o^{\text{ext}}(f)$ (*i.e.*, if $\boldsymbol{a}$ was zero). For $2\pi f\gg|\lambda_1|$ we have

$$\begin{aligned}\kappa(f) &= 1+T_*^{-1}w_1a_1\sum_{j=0}^{M-1}\frac{\epsilon^j\left(2\pi fi+|\lambda_1|\epsilon^j\right)}{(2\pi f)^2+(\lambda_1\epsilon^j)^2}\\ &\approx 1+\frac{iT_*^{-1}w_1a_1}{2\pi f}\sum_{j=0}^{M-1}\epsilon^j\\ &= 1+\frac{iT_*^{-1}w_1a_1}{2\pi f(1-\epsilon)}\end{aligned}$$

where we assumed $\left(1-\epsilon^M\right)\approx1$. Similarly, for $2\pi f\gg|\lambda_1|$,

$$\begin{aligned}H_o^{\text{ext}}(f) &= -T_*^{-1}w_1d_1\sum_{j=0}^{M-1}\frac{\epsilon^j\left(2\pi fi+|\lambda_1|\epsilon^j\right)}{(2\pi f)^2+(\lambda_1\epsilon^j)^2}\\ &\approx -\frac{iT_*^{-1}w_1d_1}{2\pi f(1-\epsilon)}\end{aligned}$$

Therefore, for $2\pi f \gg |\lambda_1|$,

$$\begin{aligned} H^{\text{ext}}(f) &= H_o^{\text{ext}}(f)/\kappa(f) \\ &\approx \frac{T_*^{-1} w_1 d_1 (1-\epsilon)^{-1}}{2\pi f i - T_*^{-1} w_1 a_1 (1-\epsilon)^{-1}}\,. \end{aligned} \tag{62}$$

For $|\lambda_1|\,\epsilon^M \ll 2\pi f \ll |\lambda_1|$, using Eq. 58 for $\mu = 0$, we obtain

$$\begin{aligned} \kappa(f) &\approx 1 + T_*^{-1} w_1 a_1 \lambda_1^{-2}\left[i\lambda_1^{-1} g(\epsilon,0)\,|\lambda_1| \ln(2\pi f/|\lambda_1|)\, g(\epsilon,-1)\right] \\ &\approx \frac{w_1 a_1 g(\epsilon,-1)}{-T_* \lambda_1} \ln(2\pi f/|\lambda_1|) \end{aligned}$$

and, similarly

$$\begin{aligned} H_o^{\text{ext}}(f) &\approx -T_*^{-1} w_1 d_1 \lambda_1^{-2}\left[i\lambda_1^{-1} g(\epsilon,0)\,|\lambda_1| \ln(2\pi f/|\lambda_1|)\, g(\epsilon,-1)\right] \\ &\approx \frac{w_1 d_1 g(\epsilon,-1)}{T_* \lambda_1} \ln(2\pi f/|\lambda_1|) \end{aligned}$$

so

$$H^{\text{ext}}(f) = H_o^{\text{ext}}(f)/\kappa(f) = -\frac{d_1}{a_1}\,.$$

Note that this expression matches with Eq. 62 for $f \to 0$.

A.7 Derivation of Eq. 44

Recall, from Eq. 41, that for $2\pi f \gg (1-\epsilon)^{-1} T_*^{-1} w_1 a$

$$H^{\text{ext}}(f) \approx -\frac{d_1}{a_1}\,. \tag{63}$$

We can simplify this expression further by substituting the expressions for a_1 and d_1. As noted in [50, , Fig. 4B] (where the $+/-/0$ notation is replaced with $H/M/L$ notation), in the HHS model $\gamma(V)$ has a high voltage threshold $\gamma_+ \gg \max(\gamma_-, \gamma_0)$ and $\delta(V)$ has a rather low voltage threshold, so it is approximately voltage independent, with

$$\delta_+ \approx \delta_- \approx \delta_0 \approx \delta_* \triangleq \delta\,. \tag{64}$$

Therefore,

$$a_1 = \tau_{\text{AP}}\left(\gamma_*(\delta_+ - \delta_-) - (\gamma_+ - \gamma_-)\delta_*\right)/(\gamma_* + \delta_*) \approx -(\gamma_+ - \gamma_-)\,\delta\tau_{\text{AP}}/(\gamma_* + \delta)$$

and

$$d_1 = (\gamma_*\delta_0 - \gamma_0\delta_*)/(\gamma_* + \delta_*) \approx (\gamma_* - \gamma_0)\,\delta/(\gamma_* + \delta)\,.$$

Finally, using

$$\gamma_* \triangleq (p_*\gamma_+ + (1-p_*)\gamma_-)\,\tau_{\text{AP}} T_*^{-1} + \left(1 - \tau_{\text{AP}} T_*^{-1}\right)\gamma_0 \approx p_*\gamma_+ \tau_{\text{AP}} T_*^{-1} + \gamma_0 \tag{65}$$

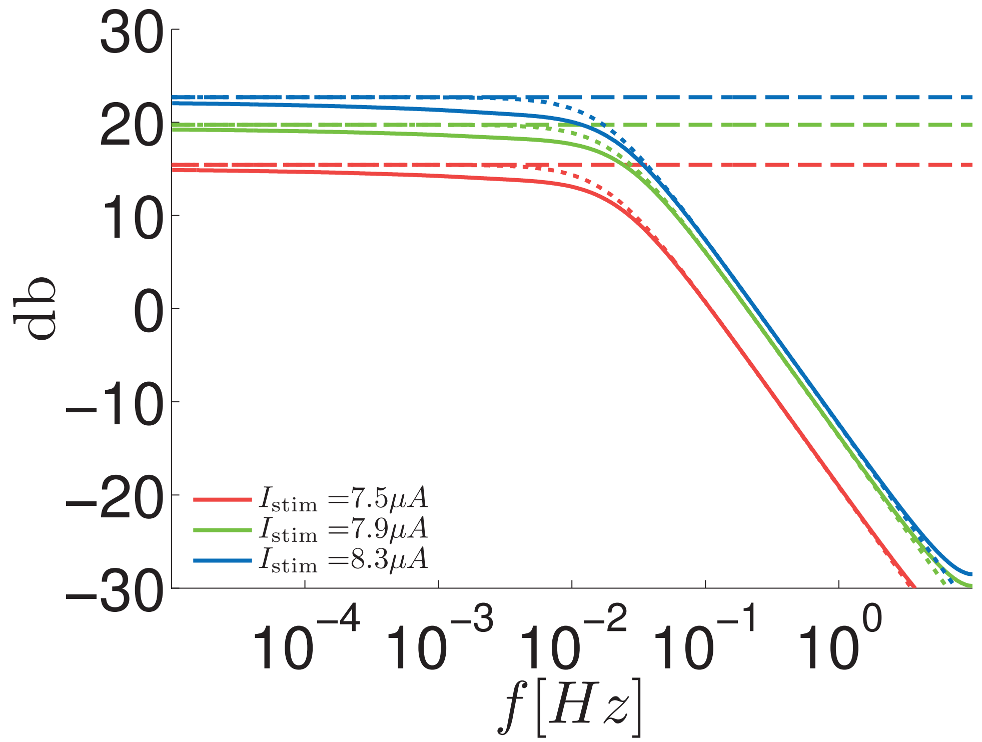

Figure 7: The external input filter $H^{\rm ext}(f)$ in the HHMS model - comparison of analytical expression (Eq. 37, solid line), asymptotic approximation (Eq. 41, dotted line) and $p_* T_*^{-1} \approx H^{\rm ext}(0)$ value (Eq. 66, dashed line).

and $\gamma_+ - \gamma_- \approx \gamma_+$, we have, for $|\lambda_1|\,\epsilon^M \ll 2\pi f \ll |\lambda_1|$,

$$-\frac{d_1}{a_1} = \frac{\gamma_* - \gamma_0}{\gamma_+ \tau_{\rm AP}} \approx p_* T_*^{-1} .$$

Therefore, in that range,

$$H^{\rm ext}(f) \approx -\frac{d_1}{a_1} \approx \frac{p_*}{T_*} . \tag{66}$$

Interestingly, we found that $H^{\rm ext}(0) \approx \bar{f}_{\rm out} \triangleq p_* T_*^{-1}$, the mean firing rate of the neuron (See Fig. 7).

B Detailed explanation of figures

B.1 Statistical measures in Fig. 3

In Fig. 3 we compare our model to the data using measures that were designed to reveal and estimate “scaling” in empirical signals, as used in [11]. These were applied exactly as in [11]. For completeness, we repeat here the details on how this was done. Further

details on meaning of these measures and the relations among them appear in [5, 25]. The measures used the count process $Z_n\left(T\right)$, generated by binning the spikes into N equally sized bins of width T. We further define the empirical average of $Z\left(T\right)$

$$\bar{Z}\left(T\right) \triangleq N^{-1}\sum_{n=1}^{N} Z_n\left(T\right) \tag{67}$$

and the empirical variance

$$\sigma_Z^2\left(T\right) \triangleq N^{-1}\sum_{n=1}^{N}\left(Z_n\left(T\right)-\bar{Z}\left(T\right)\right)^2 \tag{68}$$

If $Z_n\left(T\right)$ is a wide-sense stationary signal then for $N \to \infty$ than, from the law of large numbers, Eqs. 67 and 68 should converge to the ensemble averages $\left\langle Z_n\left(T\right)\right\rangle$ and $\left\langle \hat{Z}_n^2\left(T\right)\right\rangle$, respectively.

1. The *rate fluctuations* shows the process

$$\tilde{Z}_n\left(T\right) \triangleq Z_n\left(T\right)-\bar{Z}\left(T\right)\ ,$$

estimated using bins of different sizes ($T = 10, 30, 100$ and 300 sec) and plotted on a normalized time axis (units in number of bins).

2. The *Coefficient of Variation* (CV) is defined as the ratio of the standard deviation to the mean

$$CV\left(T\right) \triangleq \frac{\sigma_Z\left(T\right)}{\bar{Z}\left(T\right)}\ .$$

3. The *Detrended Fluctuation Analysis* (DFA) [39] was performed as follows. First, $Z_n\left(T_{\text{bin}}\right)$ was calculated with $T_{\text{bin}} = 1$ sec. Then a piecewise linear curve $U_n\left(T\right)$, with segments of length T, was fit to the $Z_n\left(T_{\text{bin}}\right)$. Then the Root Mean Square Error (RMSE) of the fit is then calculated

$$DFA\left(T\right) \triangleq \sqrt{N^{-1}\sum_{n=1}^{N}\left(Z_n\left(T_{\text{bin}}\right)-U_n\left(T\right)\right)^2}$$

4. The *Fano Factor* (FF) [25] is defined as the variance to mean ratio

$$FF\left(T\right) \triangleq \frac{\sigma_Z^2\left(T\right)}{\bar{Z}\left(T\right)}\ ,$$

5. The *Allan Factor* (AF) [25] is defined as

$$AF\left(T\right) \triangleq \frac{N^{-1}\sum_{n=1}^{N}\left(Z_n\left(T\right)-Z_{n+1}\left(T\right)\right)^2}{2\bar{Z}\left(T\right)}\ .$$

6. The firing rate *periodogram,* is an estimate of the power spectral density, as used in [11], namely

$$\tilde{S}_Z(f) \triangleq \frac{T_{\text{bin}}}{N}\left|\sum_{n=1}^{N}\tilde{Z}_n(T_{\text{bin}})\,e^{-2\pi f T_{\text{bin}} n i}\right|^2$$

with $T_{\text{bin}} = 1$ sec. Note that for $N \to \infty$, this should be a "reasonable" [5, 42] estimator for the PSD of $Z_n(T)$

$$S_Z(f) \triangleq T_{\text{bin}}\sum_{k=-\infty}^{\infty}\left\langle \hat{Z}_n(T_{\text{bin}})\,\hat{Z}_{n+k}(T_{\text{bin}})\right\rangle e^{-2\pi f T_{\text{bin}} k i} \tag{69}$$

by the Wiener-Khinchin theorem. It is straightforward to show that for periodical stimulation (*i.e.*, $T_m = T_*$), we have

$$\frac{S_Z(f)}{S_Y(f)} = \begin{cases} W & , W \le 1 \\ W\frac{\sin^2(\pi f W T_*)}{\sin^2(\pi f T_*)} & , W \ge 1 \end{cases}$$

where $S_Y(f)$ is given by Eq. 1, we denoted $W \triangleq T_*^{-1}T_{\text{bin}}$, assuming it is an integer. Note that for $f \ll T_{\text{bin}}^{-1}$ this gives

$$\frac{S_Z(f)}{S_Y(f)} \approx \begin{cases} W & , W \le 1 \\ W^3 & , W > 1 \end{cases}. \tag{70}$$

B.2 Choosing stimulation type in Fig. 5A

Suppose the stimulation is some random point-process, so $\{T_m\}$ is also a random process. One prediction we could make relates to the shape of $S_{YT}(f)$, which was not measured in [11]. Recall Eqs. 34-35. From Eq. 34, if $S_T(f)$ and $H^{\text{ext}}(f)$ are known then theoretically we can estimate $S_{YT}(f)$. Unfortunately, it is not easy to estimate $S_{YT}(f)$, due to the rather large internal fluctuations in the neuron ([48], Fig. 8). Specifically, suppose $S_{YT}^{\text{est}}(f)$ is our estimator of $S_{YT}(f)$, then the Normalized Mean Square Error (NMSE, see [4, page 321]) is

$$\text{NMSE} \triangleq \frac{\left\langle \left(S_{YT}(f) - S_{YT}^{\text{est}}(f)\right)^2\right\rangle}{\left|S_{YT}(f)\right|^2} \quad \propto \quad \frac{S_T(f)\,S_Y(f)}{\left|S_{YT}(f)\right|^2}.$$

And so, according to Eq. 35,

$$\text{NMSE} \quad \propto \quad 1 + \frac{\left|H_{\text{int}}(f)\right|^2}{\left|H^{\text{ext}}(f)\right|^2 S_T^2(f)}. \tag{71}$$

Therefore, accurate estimation of $S_{YT}(f)$ (or equivalently, identification of $H^{\text{ext}}(f)$) is harder than estimation of $S_Y(f)$ (or equivalently, identification of $H_{\text{int}}(f)$), for which the NMSE $\propto O(1)$ [4].

To overcome the estimation noise problem, we need to increase the "Signal to Noise Ratio" (SNR) in the system by increasing the "signal strength" ($S_T(f)$, the variability in T_m) in comparison with the "noise" (intrinsic fluctuations), so that

$$\left|H^{\text{ext}}(f)\right| S_T(f) \gg \left|H_{\text{int}}(f)\right|$$

in a certain range of f. In that range, we find from Eq. 71 that $\mathrm{NMSE}=O\left(1\right)$.

As indicated by Fig. 4, we expect that the best SNR would be achieved near 10^{-2}Hz. In order to test our model, we examine experimental data of a neuron under a "$f^{-\alpha}$ stimulation", taken from [12], for which $S_T\left(f\right)$ is rather large near 10^{-2}Hz, so $S_{YT}\left(f\right)$ can be estimated accurately. In Fig. 5A we see that the estimated $S_{YT}\left(f\right)$ is similar to $S_{YT}\left(f\right)$ of the fitted model with the same stimulation.

B.3 The math behind Fig. 5B

A useful method for system identification is to excite the system in all its relevant modes [16]. In our case this can be done by a sum of sinusoidal inputs. Specifically, we examine an input

$$\hat{T}_m=\sum_{l=1}^{L}T_{\mathrm{amp}}\sin\left(2\pi f_l T_* m\right) \tag{72}$$

in order to identify $H^{\mathrm{ext}}\left(f\right)$, with f_l being some positive "sample" frequencies.

From Eq. 33, the response of the neuron is given by the linear system

$$\hat{Y}\left(f\right)=H^{\mathrm{ext}}\left(f\right)\hat{T}\left(f\right)+H_{\mathrm{int}}\left(f\right)z\left(f\right).$$

Recall the response of a linear system to a sine input is a sine output, modulated by the magnitude and phase of the linear system at the frequency of the sine [34]. Therefore, in response to the input from Eq. 72,

$$\hat{Y}_m = \sum_{l=1}^{L}T_{\mathrm{amp}}\left|H^{\mathrm{ext}}\left(f_l\right)\right|\sin\left(2\pi f_l T_* m+\angle H^{\mathrm{ext}}\left(f_l\right)\right)+\mathcal{F}^{-1}\left[H_{\mathrm{int}}\left(f\right)z\left(f\right)\right].$$

The magnitudes of the sines can be found using a Discrete Fourier Transform (DFT) of $\hat{Y}_m$,

$$\hat{Y}^n\left(k\right)\triangleq\sum_{m=0}^{n-1}\hat{Y}_m e^{-2\pi kmi/n},\qquad k\in\left\{0,\ldots,n-1\right\}.$$

Since, for large n,

$$\begin{aligned}\frac{1}{n}\left|\hat{Y}^n\left(k\right)\right| &\approx \sum_{l=1}^{L}T_{\mathrm{amp}}\left|H^{\mathrm{ext}}\left(f_l\right)\right|\frac{\sin\left(\pi\left(k/n-f_lT_*\right)n\right)}{n\sin\left(\pi\left(k/n-f_lT_*\right)\right)}+\frac{1}{n}\left|H_{\mathrm{int}}\left(f\right)z\left(f\right)\right|_{f=k/T_*n}\\ &\overset{n\to\infty}{\longrightarrow} \begin{cases}T_{\mathrm{amp}}\left|H^{\mathrm{ext}}\left(f_l\right)\right| & \text{, if } k/n\approx f_lT_*\\ 0 & \text{, else}\end{cases},\end{aligned}$$

where we used the fact that $H_{\mathrm{int}}\left(f\right)$ attains its maximal values for $f\to0$ (where $H_{\mathrm{int}}\left(f\right)\sim f^{-\alpha/2}/\ln f$), and, thus, for $k>0$

$$\frac{1}{n}\left|H_{\mathrm{int}}\left(f\right)z\left(f\right)\right|_{f=k/T_*n}\le\frac{1}{n}\left|H_{\mathrm{int}}\left(f\right)z\left(f\right)\right|_{f=1/T_*n}\propto\frac{1}{n}\left|\frac{n^{\alpha/2}}{\ln\left(1/n\right)}\right|\overset{n\to\infty}{\longrightarrow}0$$

for all $\alpha\le2$. Therefore, if n is large enough, $H^{\mathrm{ext}}\left(f_l\right)$ can always be identified as peaks in $\left|\hat{Y}^n\left(k\right)\right|/nT_{\mathrm{amp}}$. In Fig. 5B we show that this method seems to work for a simulation of

the fitted HHMS model, with $T_{\text{amp}} = (0.8/L)T_*$ and $L = 9$. An experimental test of this prediction remains to be done, and would verify the linearity of the neuronal response. Note that since $LT_{\text{amp}} = 0.8T_*$, this linear response here cannot be considered simply as a "small signal response".

C Models

C.1 The HHS model

The HHS model [50] combines the Hodgkin-Huxley equations [20] with Slow sodium inactivation [6, 10]. The model equations [50], which employ the uncoupled stochastic noise approximation, are

$$
\begin{aligned}
C\dot{V} &= \bar{g}_{Na} s m^3 h \left(E_{Na} - V\right) + \bar{g}_K n^4 \left(E_K - V\right) + \bar{g}_L \left(E_L - V\right) + I\left(t\right) && (73)\\
\dot{m} &= \phi\left[\alpha_m\left(V\right)\left(1-m\right) - \beta_m\left(V\right) m\right] + \sqrt{N^{-1}\phi\left(\alpha_m\left(V\right)\left(1-m\right) + \beta_m\left(V\right) m\right)}\xi_m && (74)\\
\dot{n} &= \phi\left[\alpha_n\left(V\right)\left(1-n\right) - \beta_n\left(V\right) n\right] + \sqrt{N^{-1}\phi\left(\alpha_n\left(V\right)\left(1-n\right) + \beta_n\left(V\right) n\right)}\xi_n && (75)\\
\dot{h} &= \phi\left[\alpha_h\left(V\right)\left(1-h\right) - \beta_h\left(V\right) h\right] + \sqrt{N^{-1}\phi\left(\alpha_h\left(V\right)\left(1-h\right) + \beta_h\left(V\right) h\right)}\xi_h && (76)\\
\dot{s} &= \delta\left(V\right)\left(1-s\right) - \gamma\left(V\right) s + \sqrt{N^{-1}\left(\delta\left(V\right)\left(1-s\right) + \gamma\left(V\right) s\right)}\xi_s && (77)
\end{aligned}
$$

where V is the membrane voltage, $I\left(t\right)$ is the input current, r_j are the rapid ion channel "gating variables", s_i are the slow ion channel "gating variables", ξ are white noise processes, $\alpha\left(V\right), \beta\left(V\right), \delta\left(V\right)$, and $\gamma\left(V\right)$ are the voltage dependent kinetic rates of these gating variables, C is the membrane's capacitance, E_K, E_{Na} and E_L are ionic reversal potentials, $\bar{g}_K, \bar{g}_{Na}$ and $\bar{g}_L$ are ionic conductances, and ϕ is an auxiliary dimensionless number, and N are the number of ion channels. Most of the parameters are given their original values (as in [20, 10]):

$$
\begin{array}{ccc}
V_{Na} = 50 \text{ mV}, & V_K = -77 \text{ mV}, & V_L = -54 \text{ mV}, \\
\bar{g}_{Na} = 120\ (k\Omega \cdot cm^2)^{-1}, & \bar{g}_K = 36\ (k\Omega \cdot cm^2)^{-1}, & g_L = 0.3\ (k\Omega \cdot cm^2)^{-1},
\end{array}
$$

$$
\begin{array}{cc}
\alpha_n(V) = \frac{0.01(V+55)}{1-e^{-0.1\cdot(V+55)}} \text{ kHz}, & \beta_n(V) = 0.125 \cdot e^{-(V+65)/80} \text{ kHz}, \\
\alpha_m(V) = \frac{0.1(V+40)}{1-e^{-0.1\cdot(V+40)}} \text{ kHz}, & \beta_m(V) = 4 \cdot e^{-(V+65)/18} \text{ kHz}, \\
\alpha_h(V) = 0.07 \cdot e^{-(V+65)/20} \text{ kHz}, & \beta_h(V) = \left(e^{-0.1\cdot(V+35)} + 1\right)^{-1} \text{ kHz},
\end{array}
$$

where in all the rate functions V is used in units of mV. In order to obtain the specific spike shape and the latency transients observed in cortical neurons, some of the parameters were modified to

$$
\begin{array}{rcl}
C_m = 0.5\ \mu\text{F/cm}^2 & , & \phi = 2, \\
\gamma\left(V\right) = 0.51 \cdot (e^{-0.3\cdot(V+17)} + 1)^{-1} \text{ Hz} & , & \delta\left(V\right) = 0.05 e^{-(V+85)/30} \text{ Hz} \\
N & = & 10^6
\end{array}
$$

These specific choices were fitted to reproduce the basic experimental results of [11] on short timescales (latency transients, firing modes, firing rates and firing patterns) for certain type of neurons (loosely speaking, "non-bursting") [50].

C.2 The HHMS model

In this work we focus on the HHMS model (Hodgkin Huxley model with Many Slow variables). This model is an extension of the HHS model, in which there are many sodium currents, each with different a slow kinetic variable. The equations are identical to the HHS model, except that in Eq. 73 $\bar{g}_{Na}s$ is replaced by $\bar{g}_{Na}M^{-1}\sum_{k=1}^{M}s_k$, where s_1 has the same equation as s in the HHS model, and for $k \geq 2$,

$$\dot{s}_k = \left[\delta\left(V\right)\left(1-s_k\right) - \gamma\left(V\right)s_k\right]\epsilon^k + \sqrt{\left(\delta\left(V\right)\left(1-s_k\right) + \gamma\left(V\right)s_k\right)N_{s,k}^{-1}\epsilon^k}\xi_{s,k}\,,$$

with $\phi_{s,k} = \epsilon^k$ and $N_{s,k} = N_s\epsilon^{\nu k}$, where $\gamma\left(V\right)$ and $\delta\left(V\right)$ are taken from the HHS model. Note that N_s, ν, M and $\epsilon < 1$ are free parameters. In order to fit the experimental results in Fig. 3, we set $N_s = 10^4, \nu = 0.5, M = 5$ and $\epsilon = 0.2$ so that $S_Y\left(f\right) \sim f^{-\alpha}$, with $\alpha \approx 1.4$ (the average measured value in [11]).

D External fluctuation sources

The work in [11] investigated isolated neurons under sparse spike stimulation with fixed amplitude, as discussed here. As explained in [50], under such stimulation the neuronal response can be very sensitive to small changes in excitability - both internal or external. Therefore, it is important to make sure that the observed non-stationary $f^{-\alpha}$ behavior is not generated by external fluctuation sources that may be present in the experimental setup (an in-vitro culture of cortical neurons).

For example, such external fluctuation sources may include temperature and ion concentration fluctuations, and fluctuations in the electrode-bath interface. These are Common Non-stationary Fluctuation (CNF) sources since they should affect all the neurons that are being stimulated (in the experiments of [11], several neurons were stimulated and recorded simultaneously). Additionally, although all synaptic connections were completely blocked, there is always the possibility that some other weak interactions (*e.g.*, gap junctions, ephaptic couplings, glia cells) between different neurons might affect the results.

In this section we explain the controls performed by [11], and perform additional analysis to corroborate them. These results suggest that the $f^{-\alpha}$ behavior is independent between neurons and is therefore generated internally.

D.1 Background - experimental controls

To rule out the presence of a CNF, [11] performed two main controls. First, they included in the analyses only cells which were relatively stable during the long experiment - for which the spike profile remained stable (Fig. 5C in [11]). In these cells the latencies and response patterns remained repeatable ([11], Fig. 9). Second, they examined the correlations between different (isolated) neurons in the same experiment, and found that they were rather low (see [11], Fig. 5E). Additional basic controls have been performed, but did not appear in the paper (personal communication). For example, to rule out fluctuations in the interface electrode-baths, the stability of impedance and the shape of the stimulation pulse were tested - verifying that indeed both have not changed much from the beginning to the end of the experiment. Lastly, we comment that the culture was kept under controlled temperature of 37°c.

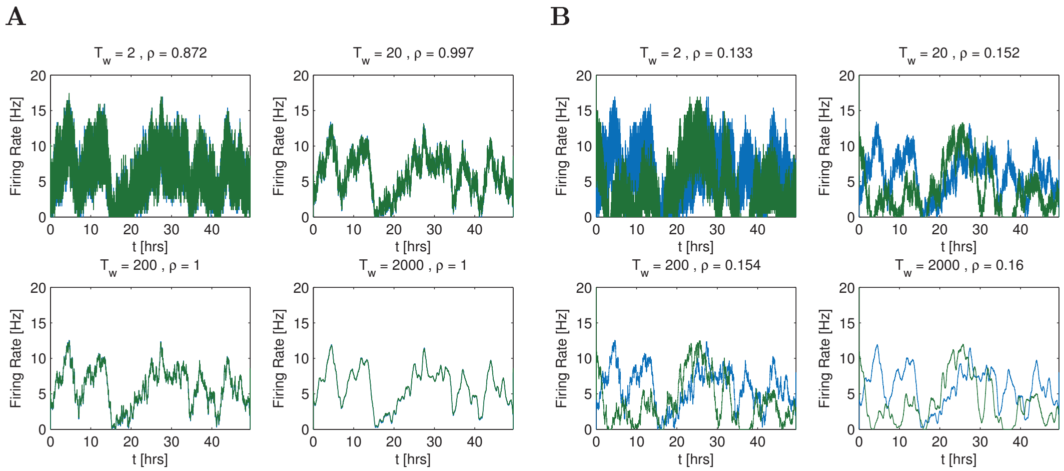

Figure 8: Firing rates for two (green and blue) simulated HHS neuron models with (**A**) common and (**B**) non-common current fluctuations in I_0, and for different averaging windows widths (T_w). Pearson correlation coefficients (see titles) are $\rho \geq 0.87$ when fluctuations are common and $\rho \sim 0.1$ when fluctuations are non-common.

D.2 Common non-stationary fluctuations

Recall that [11] found that correlations between different (isolated) neurons in the same experiment were rather low (a Pearson correlation coefficient of $\rho \sim 0.15$ or lower). If the source of the $f^{-\alpha}$ behavior in the experiment is a common noise source which affects several different neurons, one would expect these correlations to be much higher. To verify this is reasonable, we modeled the CNF as a random walk (symmetric diffusion process) in I_0, the amplitude of the injected current to the HHS model (*e.g.*, such a CNF may result from accumulating variations in the electrode interface). The step size parameter of the random walk was fitted so the magnitude of the response fluctuations would be qualitatively similar to the experimental results of [11], as can be seen in Fig. 8 (compare with Fig. 5D in [11]).

When the CNF was common (*i.e.*, the same realization of the CNF was used for two different simulations, Fig. 8A), the correlation coefficient ρ was typically very close to one, and always above 0.8 (it is not exactly one, since the model is stochastic). In contrast, when two simulations had different realizations of the CNF (*i.e.*, different sample paths, Fig. 8B) then the correlation coefficient between the firing rates in both simulations was $\rho \sim 0.15$, similarly to the experiment. Similar results were obtained when the fluctuations were added instead to the sodium conductance $\bar{g}_{\text{Na}}$ (*e.g.*, resulting from CNF in the temperature, affecting the Q10 of the channel), or to the sodium Nernst potential V_{Na} (*e.g.*, resulting from a CNF in the temperature and sodium ion concentrations).

These simulations provide a strong indication that if indeed the $f^{-\alpha}$ behavior was generated by a CNF, this would result in strong correlations between the responses of different neurons in the culture - in contrast to the low correlations observed by [11].

D.3 Inter-neuronal interactions

Recall again that in [11] the neurons are synaptically isolated. Therefore, in our work we assumed that any interaction between neurons is negligible in comparison to the intrinsic neural dynamics and stimulation, as is commonly done when modeling neurons biophysically. There is additional evidence to suggest this assumption is reasonable. First, we note that the results reported in [11] do not seem to depend on the density of the neural culture, or on the number of neurons responding to stimulation (personal communication). If these interactions were important, we would expect this not to be the case. Moreover, the correlations between different neurons are low, as we mentioned in section D.2.

However, as explained in [44], even with such low correlations, significant interactions might be present. In order to check if there might be other indications to such interactions, we performed analysis, similar to [44], on an experiment from [11], where six different neurons where simultaneously stimulated and recorded (using a time bin of 0.05 sec, as the period of the stimulation). Similarly to [44], we measured $P_{\text{emp}}(\mathbf{x})$, the empirical binary 'word' frequency observed in the data in each stimulation (*e.g.*, if, after a stimulation, the first four neurons did not respond and the last two did respond, then the resulting 'word' is $\mathbf{x} = 000011$). Then we compared the empirical word probability $P_{\text{emp}}(\mathbf{x})$ with $\prod_i P_{\text{emp}}(x_i)$, the product of the empirical probability marginals. If the neurons are completely independent then we expect both to be very similar. This is indeed the case - as can be seen in Fig. 9 (compare with fig. 2a in [44]).

This indicates that interactions between neurons in the culture in [11] are rather weak, and supports our assumption that these interactions are negligible in comparison to the intrinsic neural dynamics and stimulation.

Acknowledgments The authors are grateful to O. Barak, N. Brenner, Y. Elhanati, A. Gal, T. Knafo, Y. Kafri, S. Marom, J. Schiller and M. Ziv for insightful discussions and for reviewing parts of this manuscript. The authors are also grateful to A. Gal and S. Marom for supplying the experimental data. The research was partially funded by the Technion V.P.R. fund and by the Intel Collaborative Research Institute for Computational Intelligence (ICRI-CI).

References

[1] *http://channelpedia.epfl.ch/.*

[2] B. P. Bean, *The action potential in mammalian central neurons.*, Nature Reviews Neuroscience, 8 (2007), pp. 451–65.

[3] C. Bédard, H. Kröger, and A. Destexhe, *Does the 1/f Frequency Scaling of Brain Signals Reflect Self-Organized Critical States?*, Physical Review Letters, 97 (2006), p. 118102.

[4] J. S. Bendat and A. G. Piersol, *Random Data Analysis and Measurement Procedures*, vol. 11, Wiley, New York, NY, 3rd ed., Dec. 2000.

[5] J. Beran, *Statistics for long-memory processes*, Chapman & Hall, New York, NY, 1994.

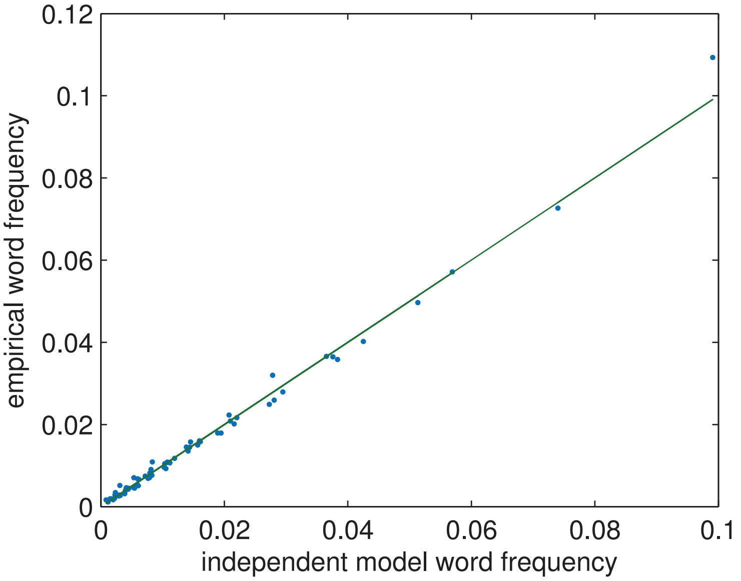

Figure 9: Binary word frequencies - empirical word frequency (the probability $P_{\text{emp}}(\mathbf{x})$) versus the independent model word frequency (a product of the marginal empirical probabilities $\prod_i P_{\text{emp}}(x_i)$) (blue dots are the words, and green line has slope one, for comparison). Both seem quite similar, indicating the the responses of different neurons are independent.

[6] W. K. Chandler and H. Meves, *Slow changes in membrane permeability and long-lasting action potentials in axons perfused with fluoride solutions*, The Journal of Physiology, 211 (1970), pp. 707–728.

[7] E. Davidson and M. Levin, *Gene regulatory networks.*, Proceedings of the National Academy of Sciences of the United States of America, 102 (2005), p. 4935.

[8] R. De Col, K. Messlinger, and R. W. Carr, *Conduction velocity is regulated by sodium channel inactivation in unmyelinated axons innervating the rat cranial meninges*, The Journal of Physiology, 586 (2008), pp. 1089–1103.

[9] D. Debanne, E. Campanac, A. Bialowas, and E. Carlier, *Axon Physiology*, Physiological Reviews, 91 (2011), pp. 555–602.

[10] I. A. Fleidervish, A. Friedman, and M. J. Gutnick, *Slow inactivation of Na+ current and slow cumulative spike adaptation in mouse and guinea-pig neocortical neurones in slices*, Journal of Physiology, 493 (1996), pp. 83–97.

[11] A. Gal, D. Eytan, A. Wallach, M. Sandler, J. Schiller, and S. Marom, *Dynamics of Excitability over Extended Timescales in Cultured Cortical Neurons*, Journal of Neuroscience, 30 (2010), pp. 16332–16342.

[12] A. Gal and S. Marom, *Entrainment of the intrinsic dynamics of single isolated neurons by natural-like input*, The Journal of Neuroscience, 33 (2013), pp. 7912–7918.

[13] A. Gal and S. Marom, *Self-organized criticality in single neuron excitability*, Physical Review E (In press), (2013).

[14] D. Gilden, T. Thornton, and M. Mallon, *1/f noise in human cognition*, Science, 267 (1995), pp. 1837–1839.

[15] D. T. Gillespie, *The chemical Langevin equation*, The Journal of Chemical Physics, 113 (2000), p. 297.

[16] G. C. Goodwin and R. L. Payne, *Dynamic system identification: Experiment design and data analysis*, Academic Press New York, New York, New York, USA, 1977.

[17] B. Y. Y. Grossman, I. Parnas, and M. E. Spira, *Differential conduction block in branches of a bifurcating axon*, The Journal of Physiology, 295 (1979), pp. 307–322.

[18] M. S. Grubb and J. Burrone, *Activity-dependent relocation of the axon initial segment fine-tunes neuronal excitability*, Nature, 465 (2010), pp. 1070–1074.

[19] B. Hille, *Ion Channels of Excitable Membranes*, Sinauer Associates, Sunderland, MA 01375, 3rd ed., 2001.

[20] A. L. Hodgkin and A. F. Huxley, *A quantitative description of membrane current and its application to conduction and excitation in nerve*, The Journal of physiology, 117 (1952), p. 500.

[21] M. S. Keshner, *Renewal process and diffusion models of 1/f noise*, PhD thesis, 1979.

[22] ———, *1/f noise*, Proceedings of the IEEE, 70 (1982), pp. 212–218.

[23] I. B. Levitan, *Modulation of ion channels by protein phosphorylation and dephosphorylation*, Annual Review of Physiology, 11 (1994), pp. 193–212.

[24] S. B. Lowen, L. S. Liebovitch, and J. A. White, *Fractal ion-channel behavior generates fractal firing patterns in neuronal models*, Physical Review E, 59 (1999), p. 5970.

[25] S. B. Lowen and M. C. Teich, *Fractal-based point processes*, 1st ed., 2005.

[26] B. N. Lundstrom, A. L. Fairhall, and M. Maravall, *Multiple timescale encoding of slowly varying whisker stimulus envelope in cortical and thalamic neurons in vivo.*, The Journal of Neuroscience, 30 (2010), pp. 5071–7.

[27] B. N. Lundstrom, M. M. H. M. H. Higgs, W. J. W. J. W. Spain, and A. L. Fairhall, *Fractional differentiation by neocortical pyramidal neurons*, Nature Neuroscience, 11 (2008), pp. 1335–42.

[28] E. Marder and J.-M. Goaillard, *Variability, compensation and homeostasis in neuron and network function.*, Nature Reviews Neuroscience, 7 (2006), pp. 563–74.

[29] S. Marom, *Neural timescales or lack thereof*, Progress in Neurobiology, 90 (2010), pp. 16–28.

[30] T. Musha and M. Yamamoto, *1/f fluctuations in biological systems*, in Proceedings of the 19th Annual International Conference of the IEEE Engineering in Medicine and Biology Society. 'Magnificent Milestones and Emerging Opportunities in Medical Engineering' (Cat. No.97CH36136), vol. 6, IEEE, pp. 2692–2697.

[31] B. Naundorf, F. Wolf, and M. Volgushev, *Unique features of action potential initiation in cortical neurons.*, Nature, 440 (2006), pp. 1060–3.

[32] M. Newman, *Power laws, Pareto distributions and Zipf's law*, Contemporary Physics, 46 (2005), pp. 323–351.

[33] H. Nishiyama, M. Fukaya, and M. Watanabe, *Axonal motility and its modulation by activity are branch-type specific in the intact adult cerebellum*, Neuron, 56 (2007), pp. 472–487.

[34] A. Oppenheim, A. Willsky, and S. Nawab, *Signals and systems*, Prentice Hall, Englewood Cliffs, NJ, 1983.

[35] P. Orio and D. Soudry, *Simple, fast and accurate implementation of the diffusion approximation algorithm for stochastic ion channels with multiple states*, PLoS ONE, 7 (2012), p. e36670.

[36] K. Padmanabhan and N. N. Urban, *Intrinsic biophysical diversity decorrelates neuronal firing while increasing information content*, Nature Neuroscience, 13 (2010), pp. 1276–1282.

[37] V. D. Paola, A. Holtmaat, G. Knott, S. Song, L. Wilbrecht, P. Caroni, K. Svoboda, and V. De Paola, *Cell type-specific structural plasticity of axonal branches and boutons in the adult neocortex.*, Neuron, 49 (2006), pp. 861–75.

[38] A. Papoulis and S. U. Pillai, *Probability, Random Variables, and Stochastic Processes*, McGraw-Hill New York, 1965.

[39] C. K. Peng, S. Havlin, H. E. Stanley, and a. L. Goldberger, *Quantification of Scaling Exponents and Crossover Phenomena in Nonstationary Heartbeat Time Series*, Chaos (Woodbury, N.Y.), 5 (1995), pp. 82–7.

[40] C. Pozzorini, R. Naud, S. Mensi, and W. Gerstner, *Temporal whitening by power-law adaptation in neocortical neurons*, Nature Neuroscience, 16 (2013), pp. 942–948.

[41] B. H. Repp, *Sensorimotor synchronization: a review of the tapping literature.*, Psychonomic bulletin & review, 12 (2005), pp. 969–992.

[42] P. M. Robinson, *Time series with long memory*, Oxford Univ Pr, New York, 2003.

[43] R. Sarpeshkar, *Analog versus digital: extrapolating from electronics to neurobiology*, Neural Computation, 10 (1998), pp. 1601–1638.

[44] E. Schneidman, M. J. Berry, R. Segev, and W. Bialek, *Weak pairwise correlations imply strongly correlated network states in a neural population.*, Nature, 440 (2006), pp. 1007–12.

[45] I. A. Silver, J. Deas, and M. Erecinska, *Ion homeostasis in brain cells: differences in intracellular ion responses to energy limitation between cultured neurons and glial cells.*, Neuroscience, 78 (1997), pp. 589–601.

[46] P. J. Sjöström, E. A. Rancz, A. Roth, and M. Häusser, *Dendritic excitability and synaptic plasticity*, Physiological Reviews, 88 (2008), pp. 769 – 840.

[47] Y. Soen and E. Braun, *Scale-invariant fluctuations at different levels of organization in developing heart cell networks.*, Physical Review E, 61 (2000), pp. R2216–9.

[48] D. Soudry and R. Meir, *The neuronal response at extended timescales: a linearized spiking input-output relation*, under review.

[49] D. Soudry and R. Meir, *History-dependent dynamics in a generic model of ion channels - an analytic study*, Front. Comput. Neurosci., 4 (2010), pp. 1–10.

[50] ——, *Conductance-based neuron models and the slow dynamics of excitability*, Front. Comput. Neurosci., 6 (2012).

[51] O. Staub, I. Gautschi, T. Ishikawa, K. Breitschopf, A. Ciechanover, L. Schild, and D. Rotin, *Regulation of stability and function of the epithelial Na+ channel (ENaC) by ubiquitination.*, The EMBO Journal, 16 (1997), pp. 6325–6336.

[52] C. Tessone, C. Mirasso, R. Toral, and J. Gunton, *Diversity-Induced Resonance*, Physical Review Letters, 97 (2006), pp. 1–4.

[53] L. Ward and P. Greenwood, *1/f noise*, Scholarpedia, 2 (2007), p. 1537.
